# The *Caenorhabditis elegans* Protein FIC-1 Is an AMPylase That Covalently Modifies Heat-Shock 70 Family Proteins, Translation Elongation Factors and Histones

**DOI:** 10.1371/journal.pgen.1006023

**Published:** 2016-05-03

**Authors:** Matthias C. Truttmann, Victor E. Cruz, Xuanzong Guo, Christoph Engert, Thomas U. Schwartz, Hidde L. Ploegh

**Affiliations:** 1 Whitehead Institute for Biomedical Research, Cambridge, Massachusetts, United States of America; 2 Department of Biology, Massachusetts Institute of Technology (MIT), Cambridge, Massachusetts, United States of America; Genentech, UNITED STATES

## Abstract

Protein AMPylation by Fic domain-containing proteins (Fic proteins) is an ancient and conserved post-translational modification of mostly unexplored significance. Here we characterize the *Caenorhabditis elegans* Fic protein FIC-1 *in vitro* and *in vivo*. FIC-1 is an AMPylase that localizes to the nuclear surface and modifies core histones H2 and H3 as well as heat shock protein 70 family members and translation elongation factors. The three-dimensional structure of FIC-1 is similar to that of its human ortholog, HYPE, with 38% sequence identity. We identify a link between FIC-1-mediated AMPylation and susceptibility to the pathogen *Pseudomonas aeruginosa*, establishing a connection between AMPylation and innate immunity in *C*. *elegans*.

## Author Summary

Eukaryotic Fic domain containing proteins (Fic proteins) AMPylate target proteins at the expense of a single ATP molecule. Previous studies have established a first link between target protein AMPylation and the unfolded protein response (UPR) in the endoplasmic reticulum. Yet, the consequences of target AMPylation remain poorly understood. Here, we take a multi-faceted approach to investigate the role of the *C*. *elegans* Fic protein FIC-1 on a biochemical, structural and functional level *in vitro* as well as *in vivo*. We solve the 3-dimensional structure of FIC-1 and identify novel FIC-1 substrates belonging to the translation elongation as well as heat-shock protein families. Investigating the consequence of diminished (*fic*-*1*(n5823)) or increased (FIC-1[E274G](nIs733)) AMPylation levels *in vivo*, we find a link between AMPylation and the innate immune response to the bacterial pathogen *P*. *aeruginosa*, describing a novel in vivo phenotype associated with Fic protein mediated target AMPylation.

## Introduction

How post-translational modifications regulate protein activity is a fundamental question in biology. Phosphorylation, methylation or acetylation reversibly control cellular signaling pathways, the misregulation of which is often associated with pathologies, including cancer or autoimmune diseases [1, 2]. AMPylation, the covalent addition of AMP to a target protein, has recently been described as a new post-translational modification found in both prokaryotes and eukaryotes. However, protein AMPylation is far less well understood as a post-translational modification and its implications for cellular physiology remain largely unknown.

Protein AMPylation in metazoans is catalyzed by fic-domain containing proteins (Fic proteins). Fic proteins are an evolutionarily conserved protein family, numbering approximately 2700 members distributed over most kingdoms of life, with the exception of fungi and plants [3, 4]. While many bacterial species encode a number of different Fic proteins, most eukaryotes,—including *Caenorhabditis elegans*, *Drosophila melanogaster*, *Mus musculus* and *Homo sapiens*—carry only a single gene that specifies a Fic family member. All Fic proteins share a conserved sequence motif (HxFx(D/E)GN(G/K)R) found in their respective fic domains, including an invariant histidine required for catalysis [5]. Fic proteins accept a variety of nucleotide substrates, including ATP and UTP, to covalently AMPylate (adenylylate), UMPylate or phosphorylate their targets. However, protein AMPylation—the covalent addition of an AMP moiety to the target protein at the expense of a single ATP—is their predominant activity [6–9]. In bacteria, Fic protein-mediated AMPylation of Gyrase and Topoisomerase IV has been linked to toxin-antitoxin systems such as the VbhT-VbhA pair found in *Bartonella schoenbuchensis* [5, 10]. In addition, several pathogens evolved effector proteins equipped with Fic-domains that—upon translocation into the host cell—interfere with host cell signaling. They do so by covalently AMPylating and thus inactivating small GTPases of the Rho and Rab family [11, 12].

In eukaryotes, AMPylation by Fic proteins may regulate the unfolded protein response (UPR), as well as carry out covalent modification of histones [13–16]. The Drosophila Fic protein dfic as well as the human Fic protein HYPE target and modify the ER-resident HSP70 protein BiP/GRP78 [14, 15]. While ER stress increases intracellular dFic / HYPE as well as BiP levels, the consequences of ER stress for AMPylation and activity of BiP remains controversial. Induction of the UPR may lessen BiP AMPylation, whereas a competing model infers an increase in AMPylation of BiP, resulting in increased ATPase activity [14–16]. Addressing Fic protein biology in *C*. *elegans* may help to resolve this paradox.

Here, we characterize the *C*. *elegans* Fic protein FIC-1 *in vitro* and *in vivo*. We show that FIC-1 modulates antimicrobial defense responses of *C*. *elegans* against *Pseudomonas aeruginosa*, often used as a simple eukaryotic model of infectious disease and innate immunity. We show that FIC-1 is ubiquitously expressed throughout the nematode body. We demonstrate that FIC-1 acts as an AMPylase and covalently modifies core histones, HSP 70 family members and translation elongation factors. Finally, we determine the crystal structure of FIC-1 and its constitutively active mutant form FIC-1 E274G and identify a potential binding site for endogenous regulators. Our results provide the first evidence for a role of Fic protein-mediated AMPylation in protection of the host.

## Results

### FIC-1 alters susceptibility to killing by *P*. *aeruginosa*

**Fig 1:**
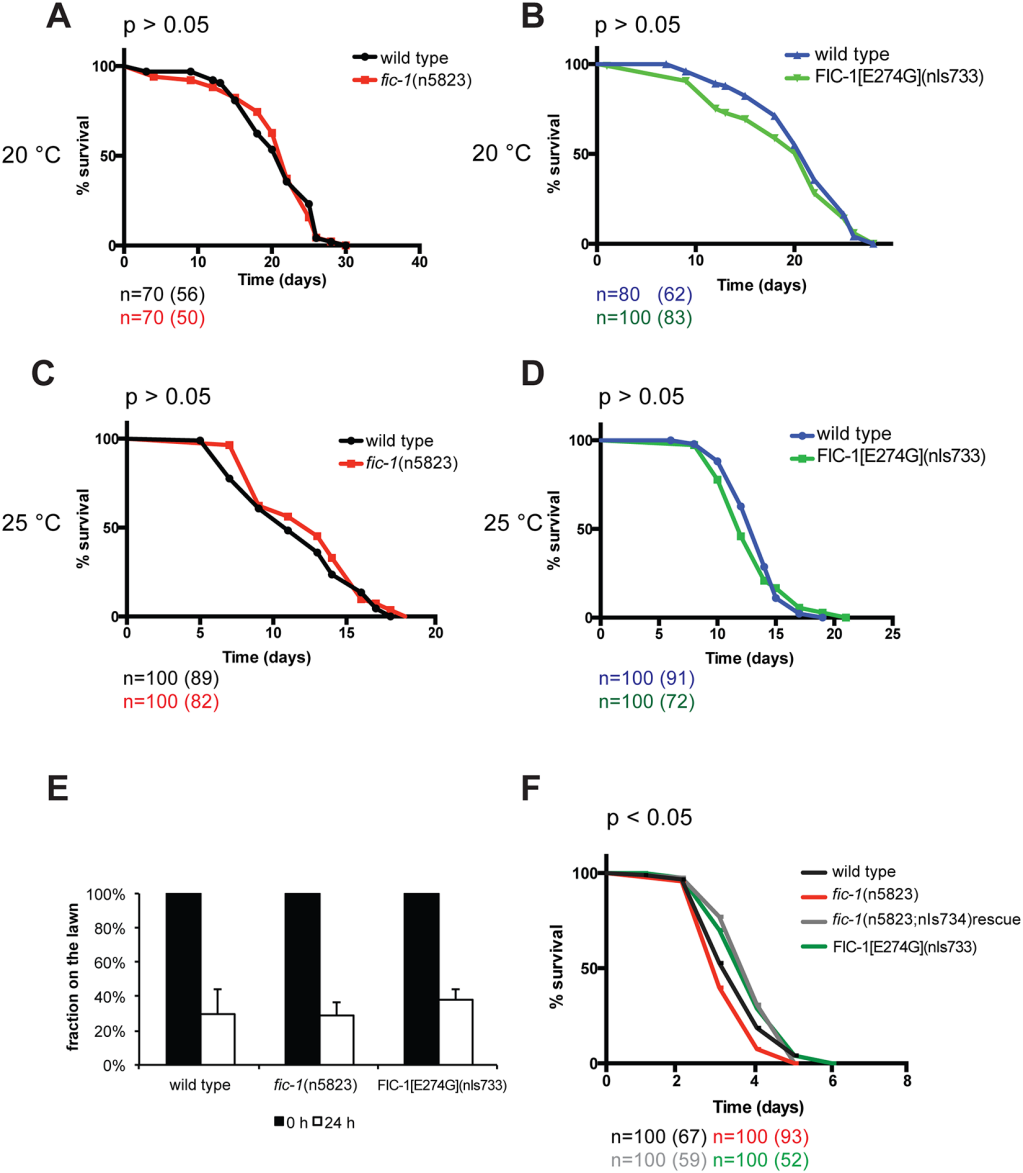
AMPylation plays a role in susceptibility to *P*. *aeruginosa* infections. AMPylation levels have no influence on aging: wild type, *fic-1*(n5823) and FIC-1[E274G](nIs733) animals were kept at either 20 C (A and B) or 25 (C and D) and survival was scored every other day. Depicted n refers to number of animals at experiment initiation; number in brackets represents total counted dead events. (E) AMPylation has no consequences on pathogen avoidance: L4 nematodes were placed in the center of a *P*. *aeruginosa* loan and animal localization was scored after 24 hours. (F) FIC-1[E274G](nIs733) increases while *fic-1*(n5823) decreases pathogen tolerance: L4 animals were place in the center of a *P*. *aeruginosa* loan and nematode survival was scored once per day until last animal vanished. Depicted n refers to number of animals at experiment initiation; number in brackets represents total counted dead events. Representative replica shown. P-values (Gehan-Breslow-Wilcoxon test) as compared to N2 wild type control: N2. vs *fic-1*(n5823): 0.046; N2 vs. *fic-1*(n5823, nIs734) rescue: 0.009; N2 vs. FIC-1[E274G](nIs733): 0.042; *fic-1*(n5823) vs. *fic-1*(n5823, nIs734) rescue or FIC-1[E274G](nIs733): <0.0001. Additional independent replica are depicted in S7 Fig.

*C*. *elegans*, which carries a single gene encoding a fic-domain containing protein (FIC-1), is a versatile model to study longevity, stress responses or innate immunity. We asked if changes in FIC-1 levels or activity resulted in global fitness defects in *C*. *elegans*. Therefore we created a *fic-1* deletion allele using CRISPR technology. The deletion allele (*n5823*) contains a 7 bp deletion in *fic-1*’s Exon IV, resulting in a pre-mature stop codon (S1A and S1B Fig). We also expressed a presumably constitutively active form of *fic-1*, FIC-1[E274G](nIs733) under the control of its endogenous promotor. First, we performed longevity assays to evaluate whether changes in FIC-1 activity might affect lifespan and observed no significant differences between *fic*-1(n5823) mutants, and FIC-1[E274G](nIs733) constitutively active animals or wild type controls at 20°C or 25°C (Fig 1A–1D, additional independent replicate shown in S8A–S8C Fig). When grown on a *P*. *aeruginosa* lawn, however, *fic*-1(n5823) mutants displayed increased susceptibility to killing by *P*. *aeruginosa* as compared to wild type (Fig 1F, additional independent replicate shown in S9A–S9H Fig). This was not due to a defect in pathogen sensing, as *fic-1*(n5823) mutant animals appeared to be unaffected in avoidance assays (Fig 1E). Expression of FIC-1 E274G slightly increased pathogen tolerance and enhanced relative infection outcome. Further, we could rescue survival of *fic-1*(n5823) mutants by expressing wild type *fic-1* in the mutant animals. Together, these results establishing a role for Fic proteins in innate immunity.

### FIC-1 is not a master regulator of ER stress responses in *C*. *elegans*

Studies in *D*. *melanogaster* as well as in human cells suggested a connection between Fic proteins and the regulation of the unfolded protein response (UPR) [14–16]. In *C*.*elegans*, animal development on *P*. *aeruginosa* requires the presence of the X-box binding protein 1 (XBP-1), an immediate downstream target of the IRE-1-regulated UPR branch, while other UPR branches were dispensable [17].

**Fig 2:**
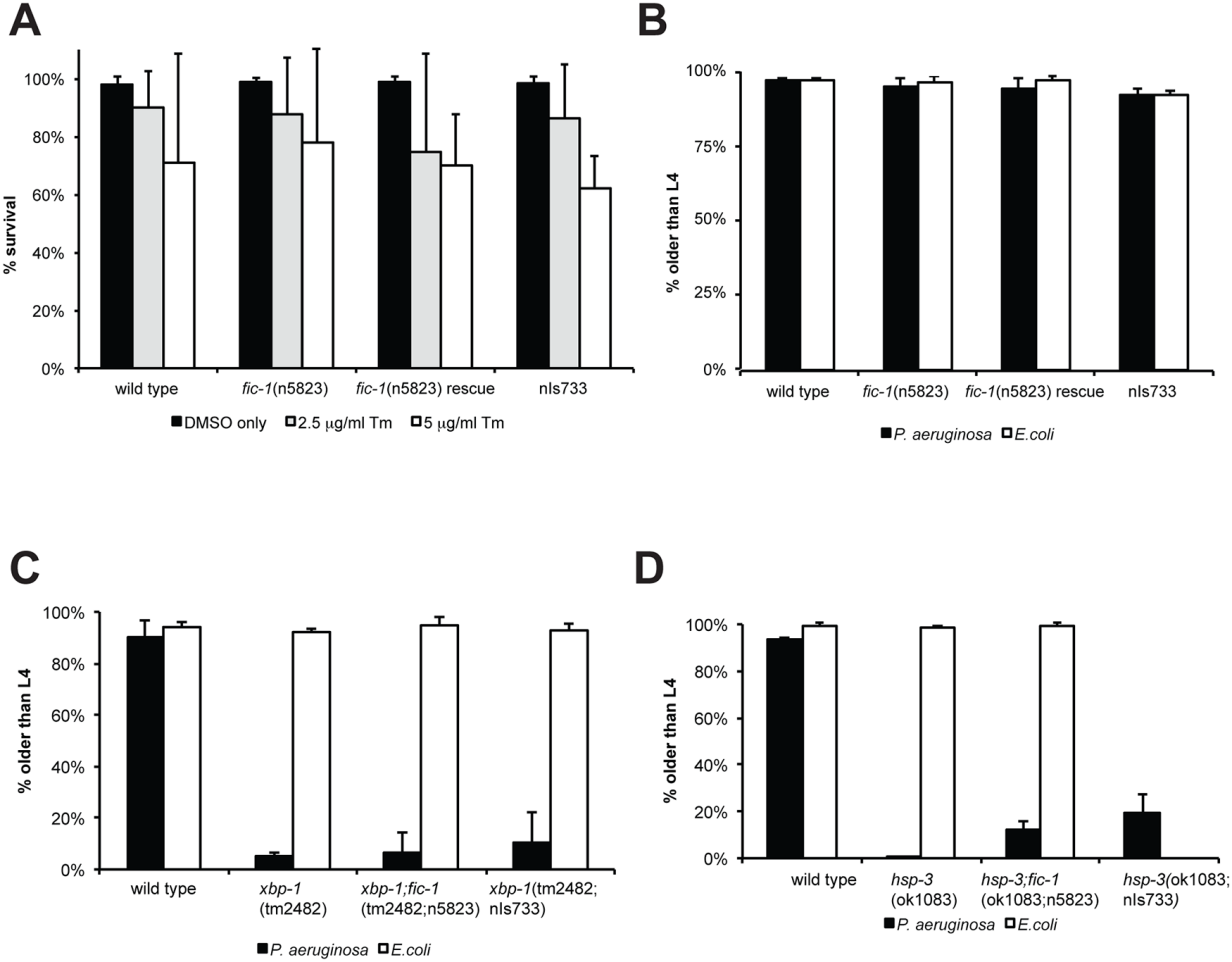
Hyper- or hypo-AMPylation has no apparent consequences on nematode viability and response to acute or chronic ER stress. (A) AMPylation has no influence on development under acute ER stress: eggs were transferred to OP50 plates containing different concentrations of tunicamycin to induce acute ER stress. Embryo development was scored. Average of three independent experiments shown here. (B) AMPylation has no influence on development under chronic ER stress: eggs were transferred to *P*. *aeruginosa* plates to induce chronic ER stress. Embryo development was scored. Average of three independent experiments shown here. (C) and (D) development assay under chronic ER stress: eggs of indicated lines were transferred to *P*. *aeruginosa* plates to induce chronic ER stress. Embryo development was scored. Average of three independent experiments shown here.

To investigate if the observed *fic*-*1*-dependent changes in pathogen susceptibility were linked to changes in ER stress signaling, we examined involvement of *fic-1* in the induction of stress responses upon exposure to specific stress cues. The reporter constructs *hsp-4*::GFP (ER stress) or *hsp-6*::GFP (mitochondrial stress) revealed no apparent difference in either the ER (tunicamycin) or mitochondrial (ethidium bromide) stress response when tested in *fic-1*(n5823) mutants or animals expressing FIC-1[E274G](nIs733) (S2A and S2B Fig), using *E*.*coli* as a food source. When we transferred embryos onto NGM plates containing various concentrations of tunicamycin and scored development as well as adult survival, *fic-1*(n5823) loss of function nor the constitutively active form (nIs733) affected the outcomes (Fig 2A). Thus, we conclude that FIC-1 is not essential in inducing or sustaining the UPR in *C*. *elegans*. Next, we repeated our development assays, exposing animals to *Pseudomonas aeruginosa*. Changes in FIC-1 activity did not affect nematode development on *P*. *aeruginosa* (Fig 2B). As *C*. *elegans* encodes two Grp78/BiP homologues, *hsp-3* and *hsp-4*, assumed to cross-compensate for each other in their roles as ER-residing protein chaperones, we repeated these assays in the presence of *P*. *aeruginosa* or tunicamycin in *fic-1;hsp-3* mutants to possibly render them more sensitive to changes in FIC-1 activity. We also examined the impact of FIC-1 in a *xbp-1*-deficient background, because in human cells the xbp-1-linked branch of the UPR is not known to be modulated by HYPE, while the PEK-1 and ATF-6-linked branches are [14]. None of the conditions tested showed a significant difference between *fic-1*(n5823) mutants or constitutive active (nIs733) animals and their respective controls (Fig 2C and 2D, S2C and S2D Fig). *fic-1* is therefore not a key regulator of the UPR in *C*. *elegans*. Of note, *hsp-3* nematodes were very sensitive to *P*. *aeruginosa* exposure during early development and only few reached adulthood, highlighting a role for HSP-3 in the tolerance of chronic ER stress and innate immunity.

### FIC-1 is expressed throughout *C*. *elegans* and enriched at the nuclear interface

**Fig 3:**
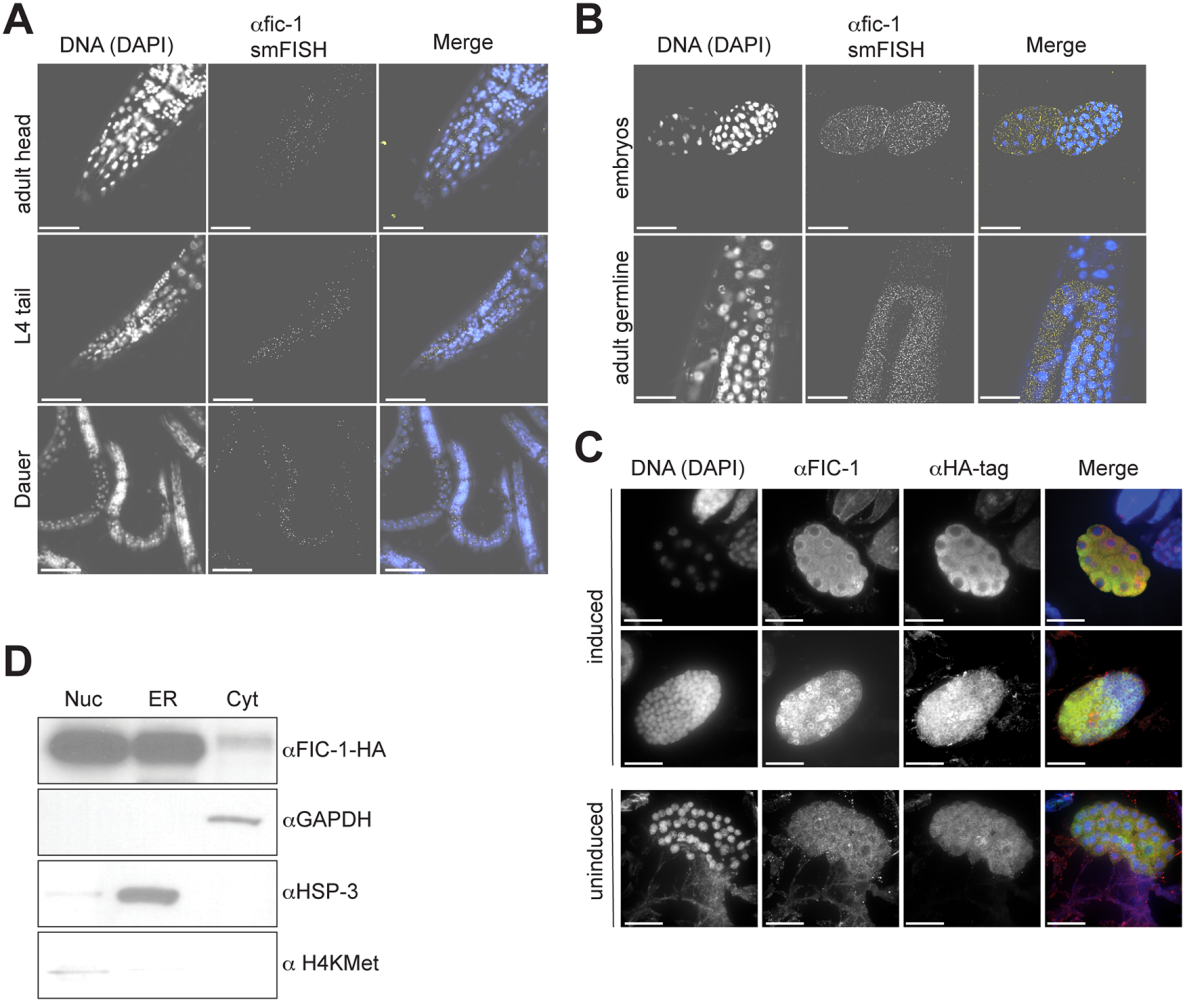
FIC-1 is enriched in the adult germline as well as nematode embryos and localizes to the nuclear membrane. (A) FIC-1 is expressed ubiquitously albeit at low levels: FIC-1 expression pattern as detected by smFISH analysis; samples stained with DAPI (left panel) and smFISH probe (middle panel). Right panel shows merged image where nuclei are represented in blue and smFISH signal in yellow. Distinct representative developmental stages shown here (B) FIC-1 is enriched in the adult germline and embryos: FIC-1 expression pattern as detected by smFISH analysis; samples stained with DAPI (left panel) and smFISH probe (middle panel). Right panel shows merged image where nuclei are represented in blue and smFISH signal in yellow. (C) FIC-1 preferentially localizes to the nuclear envelope/ER: embryos over-expressing HA-tagged FIC-1 were stained with indicated antibodies and dyes and FIC-1 localization was analyzed by confocal microscopy. (D) FIC-1 preferentially localizes to the nuclear envelope/ ER: sub-cellular fraction of *C*. *elegans* embryos. Individual fractions probed with indicated antibodies for enrichment of tested proteins. Nuc: nuclear fraction; ER: ER fraction; Cyt: cytosolic fraction.

To get a better understanding of the cell types and body parts that express FIC-1 *in vivo*, we applied a single molecule fluorescence *in situ* hybridization assay (smFISH) to detect endogenous *fic-1* mRNA [18]. Our results showed the presence of *fic-1* mRNA throughout the animal’s body, including the germline, and at all developmental stages (Fig 3A and 3B). Embryonic expression levels appeared comparatively high and were further confirmed in animals expressing GFP under the control of the putative *fic-1* promotor (P*fic-1*::*gfp*) (S3A Fig). To characterize localization of FIC-1, we analyzed by fluorescence microscopy embryos of animals that express C-terminally HA-tagged FIC-1 under the control of a strong heat-shock promotor. Inducible FIC-1 expression was confirmed by immunoblotting (S3B Fig), and FIC-1-HA was detected using either an anti-HA antibody or a mouse anti-FIC-1 serum previously validated using recombinant FIC-1 proteins (S3C Fig). We detected multiple FIC-1-HA species by immunoblotting with anti-HA antibody in *C*.*elegans* lysates, ranging in size from 58 kDa (full length) to approximately 30 kDa, indicating N-terminal processing of a fraction of FIC-1 (S3C Fig) either by proteolysis or alternative translation initiation. While we were unable to detect FIC-1 prior to induction (Fig 3C, uninduced), we observed low levels of FIC-1 expression throughout the cell with a notable accumulation of FIC-1 at the nuclear interface or the nuclear envelope / ER upon heat-shock (Fig 3C, induced). This enrichment at the nucleus-ER interface is reminiscent of intracellular HYPE localization [13, 14]. We further probed FIC-1 localization by sub-cellular fractionation and immunoblotting and confirmed significant enrichment of FIC-1 in the nuclear and ER fractions, with less presence in the cytoplasm (Fig 3D). We also over-expressed GFP-tagged FIC-1 E274G in HeLa cells and analyzed its intracellular distribution pattern by microscopy. We likewise observed GFP-FIC-1 accumulation in the nuclear envelope as well as in associated structures, similar to the pattern seen for GFP-HYPE E234G (S3D Fig). We conclude that FIC-1 is ubiquitously expressed in *C*. *elegans* and shows an intracellular localization pattern similar to that of HYPE, with an additional presence of FIC-1 in the cytoplasm.

### FIC-1 is an AMPylase

**Fig 4:**
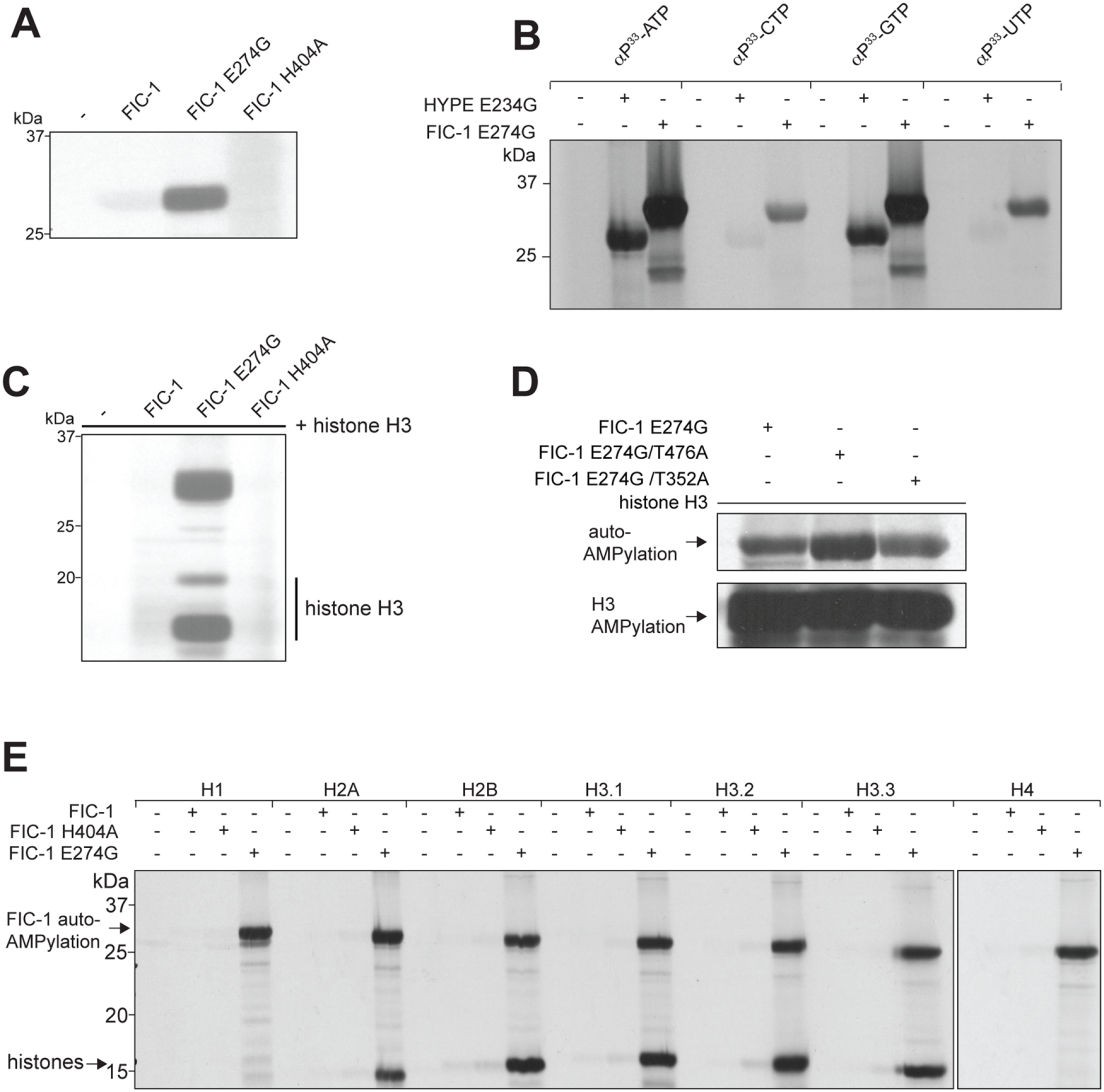
FIC-1 is an AMPylase. (A) FIC-1 exhibits auto-AMPylation activity: Recombinant FIC-1, FIC-1 E274G or FIC-1 H404A was incubated with α ^33^P-ATP for an hour and incorporation of label was assessed by SDS-PAGE and autoradiography. (B) FIC-1 accepts different nucleotide substrates: FIC-1 E274G or HYPE E234G were incubated with respective α ^33^P-labeled nucleotides for one hour at room temperature and sample autoradiography was assessed. (C) FIC-1 E274G AMPylates histone H3: Recombinant FIC-1, FIC-1 E274G or FIC-1 H404A was incubated with α ^33^P-ATP for an hour at which point histone H3 was added and the mixture was incubated for an additional hour. Incorporation of label was assessed by SDS-PAGE and autoradiography. (D) FIC-1 E274G/T476A and FIC-1 E274G/T352A are fully active: Recombinant FIC-1 E274G, FIC-1 E274G/T476A and FIC-1 E274G/T352A was incubated with α ^33^P-ATP for an hour at which point histone H3 was added and the mixture was incubated for an additional hour. Incorporation of label was assessed by SDS-PAGE and autoradiography. (E) FIC-1 AMPylates core histones H2 and H3 but not H4: Recombinant FIC-1, FIC-1 E274G or FIC-1 H404A was incubated with α ^33^P-ATP for an hour at which point purified histone substrates were added and the mixture was incubated for an additional hour. Incorporation of label was assessed by SDS-PAGE and autoradiography.

To test a possible catalytic activity of FIC-1, we expressed and purified from *E*. *coli* a truncated version of FIC-1, as well as two additional mutant versions, FIC-1 E274G and FIC-1 H404A. While substituting the corresponding glutamic acid in FIC-1’s human ortholog HYPE with glycine results in a hyper-active enzyme (E234G), exchanging the conserved histidine with an alanine (H363A) diminishes HYPE’s target AMPylation activity. We first assessed self-AMPylation using αP^33^-ATP (Fig 4A). As expected, wild type FIC-1 showed only weak self-AMPylation, FIC-1 E274G exhibited a massive increase in self-modification, while FIC-1 H404A did not show detectable self-AMPylation. We also tested the ability of FIC-1 E274G to accept nucleotides other than ATP as a substrate for self-modification (Fig 4B). Self-AMPylation as well as self-GMPylation proceeded at high rates, but we also observed self-CMPylation and self-UTPylation, indicating that FIC-1 E274G is even more promiscuous in its preference for nucleoside triphosphates than HYPE E234G. To test for target AMPylation, we repeated our FIC-1 *in vitro* AMPylation assay using recombinant histone H3 as substrate (Fig 4C). FIC-1 as well as FIC-1 E274G AMPylated histone H3 while FIC-1 H404A showed no such activity. To map the modified residue(s) on FIC-1, we performed self-AMPylation assays using ATP and subjected the samples to LC-MS/MS. We identified two auto-AMPylation sites, T352 and T476, AMPylated in FIC-1_258−508_, FIC-1_258−508_ E274G, FIC-1_134−508_ and FIC-1_134−508_ E274G samples (S4A and S4B Fig). Mutation of these sites individually in a FIC-1 E274G background did not drastically affect auto- or target AMPylation levels (Fig 4D). We also explored the ability of FIC-1 to AMPylate other histone family proteins previously identified as targets for its human homologue HYPE [13]. FIC-1 E274G modified histones H2A, H2B, H3.1, H3.2 H3.3 but not H1 or H4. Thus, the set of *in vitro* FIC-1 targets overlaps greatly with that of HYPE, yet differs at least in its ability to modify histone H4 (Fig 4E).

### Structure and conservation of FIC-1

**Fig 5:**
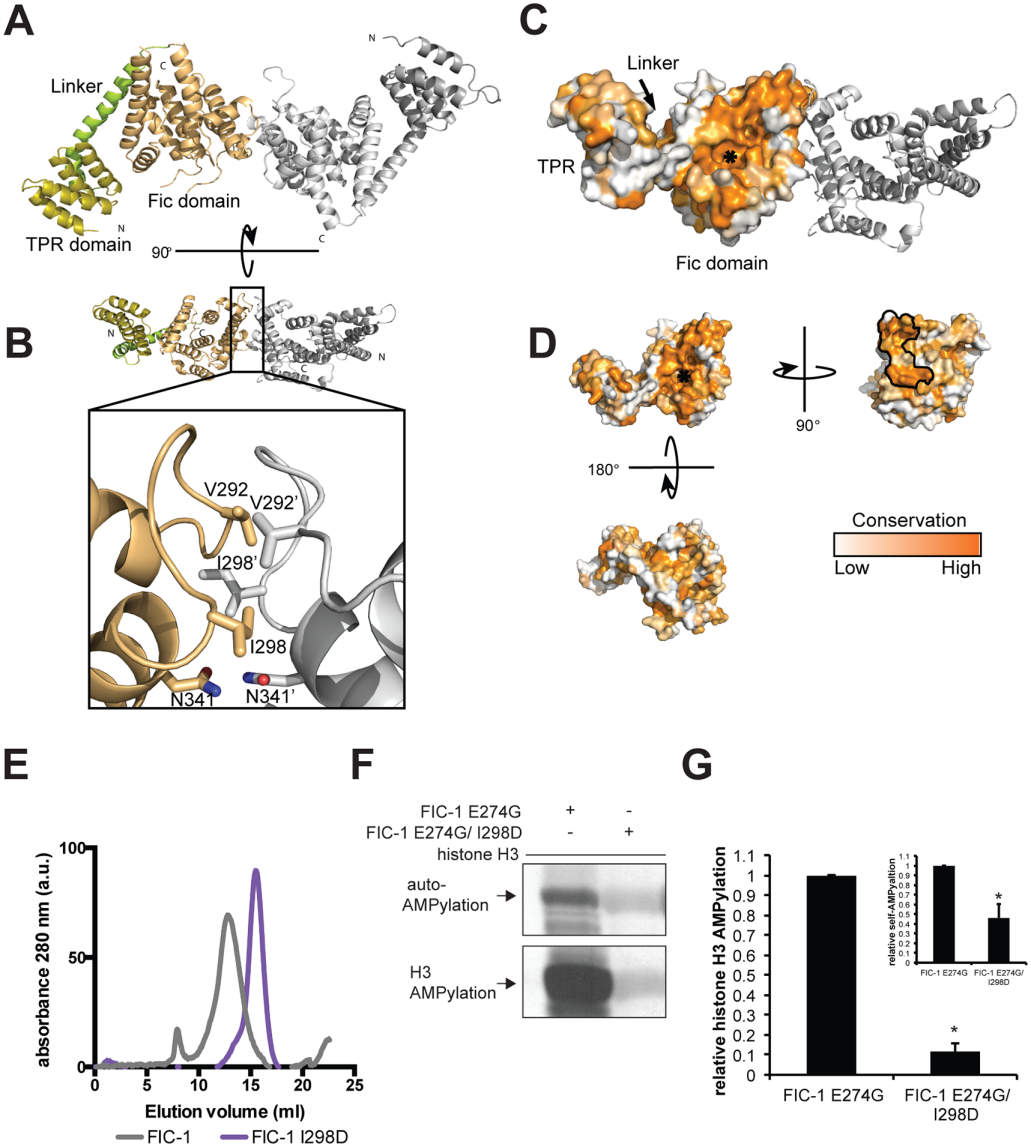
FIC-1 structure, domains and dimer interface. (A) Ribbon representation of FIC-1 dimer, with individual domains colored in a single monomer. (B) Cartoon representation of FIC-1; the dimer interface is highlighted in the inset where key side chain and backbone contacts are shown. (C) Surface representation of FIC-1 monomer and ribbon representation of a second monomer, the ATP binding site is highlighted with a white asterisk. (D) Surface representation of FIC-1 monomer; coloring is based on conservation from an alignment between Fic-domain containing proteins. Dimerization interface is outlined in black. (E) Size exclusion chromatogram showing elution profiles of FIC-1 wildtype (wt), and FIC-1 I298D. Elution volumes of standards are highlighted with arrows. (F) Monomeric FIC-1 E274G/I298D exposes reduced AMPylation activity: FIC-1 E274G or FIC-1 E274G/I298D were pre-incubated with α ^33^P-ATP for an hour before histone H3 was added and the mixture was incubated for another hour. Incorporation of label was assessed by SDS-PAGE and autoradiography. (G) Quantification of histone H3 and self-AMPylation (inlet). Data shown represents the average of two independent replica. * = p-value < 0.01 (t-test).

The crystal structure of HYPE was described recently [19]. The differences in target specificity of HYPE and FIC-1 as well as their sequence divergence (38% amino acid sequence identity) prompted us to solve the structures of FIC-1 and FIC-1 E274G crystallographically. Both proteins crystallized in the same space group and the structures could readily be solved by molecular replacement using apo-HYPE as a search model. The asymmetric unit contains a dimer of FIC-1, arranged in a similar way as seen with human HYPE (Fig 5A and S5A and S5B Fig). Like HYPE, FIC-1 is also a tripartite, entirely helical protein, with an N-terminal TPR element followed by a linker helix connecting to the Fic domain. The TPR element contains two stacked TPR motifs, each composed of two antiparallel helices. The fic domain of FIC-1 and HYPE superpose very well with a root mean square deviation of 1.4 Å. Only the linker helix and the TPR element are slightly shifted with respect to another, likely influenced by the different crystal packing environments. The fic domain in FIC-1 consists of 8 α-helices in total, where three helices precede the fic core and the ATP binding site. The first of these helices, known as “α-inh”, contains the auto-inhibitory glutamate at position 274, while the following two helices have been dubbed the pre-A and pre-B helices. The fic core itself is a four-helix bundle (α1-α4), containing the conserved catalytic motif (HxFx(D/E)(A/G)N(GK)R), represented in *C*. *elegans* by the sequence **H**P**F**T**DGNGR** [20]. The next conserved feature is a loop located between helices 2 and 3 of the fic core, called the flap. The flap is not visible in our structure, which suggests inherent flexibility of this motif. After the fic core, the final two helices called helices post-A and post-B pack against both the TPR motif and the linker helix, positioning the C terminus close to the loop between the linker helix and the auto inhibitory helix (S5C Fig, top panels). FIC-1 crystallized with a sulfate occupying the β-phosphate site of ATP, to highlight similar features with HYPE we modeled an ATP molecule onto our Fic-1 structure (S5C Fig, bottom panels), the resulting hydrogen-bonding pattern between the fic core of FIC-1 and ATP are nearly identical to that of HYPE and ATP. Our data also suggest that eukaryotic Fic proteins are constitutive dimers.

Similar to the human fic domain-containing protein HYPE, FIC-1 also crystallizes as a dimer. The dimer is held together by two discrete interfaces that together bury 676.4 Å^2^ of solvent-accessible surface area (Fig 5B). The first interface is more extensive (384.9 Å^2^) and is composed of helix pre-B and its preceding loop. This interaction is likely driven by the hydrophobic effect of burying V292 and I298 of both monomers in the nearly symmetrical binding interface. In the second interface (291.4 Å^2^) the hydrogen bonding between the side chains of N341 is the most noticeable feature (Fig 5B, inset).

To test if FIC-1 is a dimer in solution and if dimerization is required for enzymatic activity, we generated point mutants designed to disrupt the dimerization interface. Based on our structure we predicted that an I298D mutation should inhibit dimer formation without affecting the protein fold. We cloned and purified FIC-1 I298D as well as FIC-1 E274G/I298D. As expected, the mutant protein behaved as a monomer in solution as shown by size exclusion chromatography while FIC-1 wild-type eluted significantly earlier (Fig 5E). *In vitro* AMPylation testing FIC-1 E274G/I298D indicated a direct connection between FIC-1 dimerization and its ability to modify targets: Relative to FIC-1 E274G’s capacity to AMPylate histone H3, FIC-1 E274G/I298D self-AMPylation was reduced to 46% (Fig 5F, upper panel and Fig 5G) and target AMPylation activity to 11% (Fig 5F, lower panel and Fig 5G), respectively.

To further compare FIC-1 with other eukaryotic Fic representatives, we performed an alignment of fic domain-containing proteins in highly divergent metazoans and mapped the conserved features to the surface of a FIC-1 monomer (Fig 5C). As expected, the ATP binding site is conserved throughout, while the TPR domain and the linker helix show less conservation. The dimer interface itself is highly conserved, suggesting that the FIC-1 dimer is the active form of the proteins in metazoans (Fig 5D). On the opposite side from the ATP-binding pocket, we observe another conserved region that forms a deep groove (Fig 5D, bottom left). This groove may accommodate binding partners, or provide an assembly point for (a) larger complex(es).

### FIC-1 AMPylates heat-shock protein 70 family members as well as translation elongation factors

**Fig 6:**
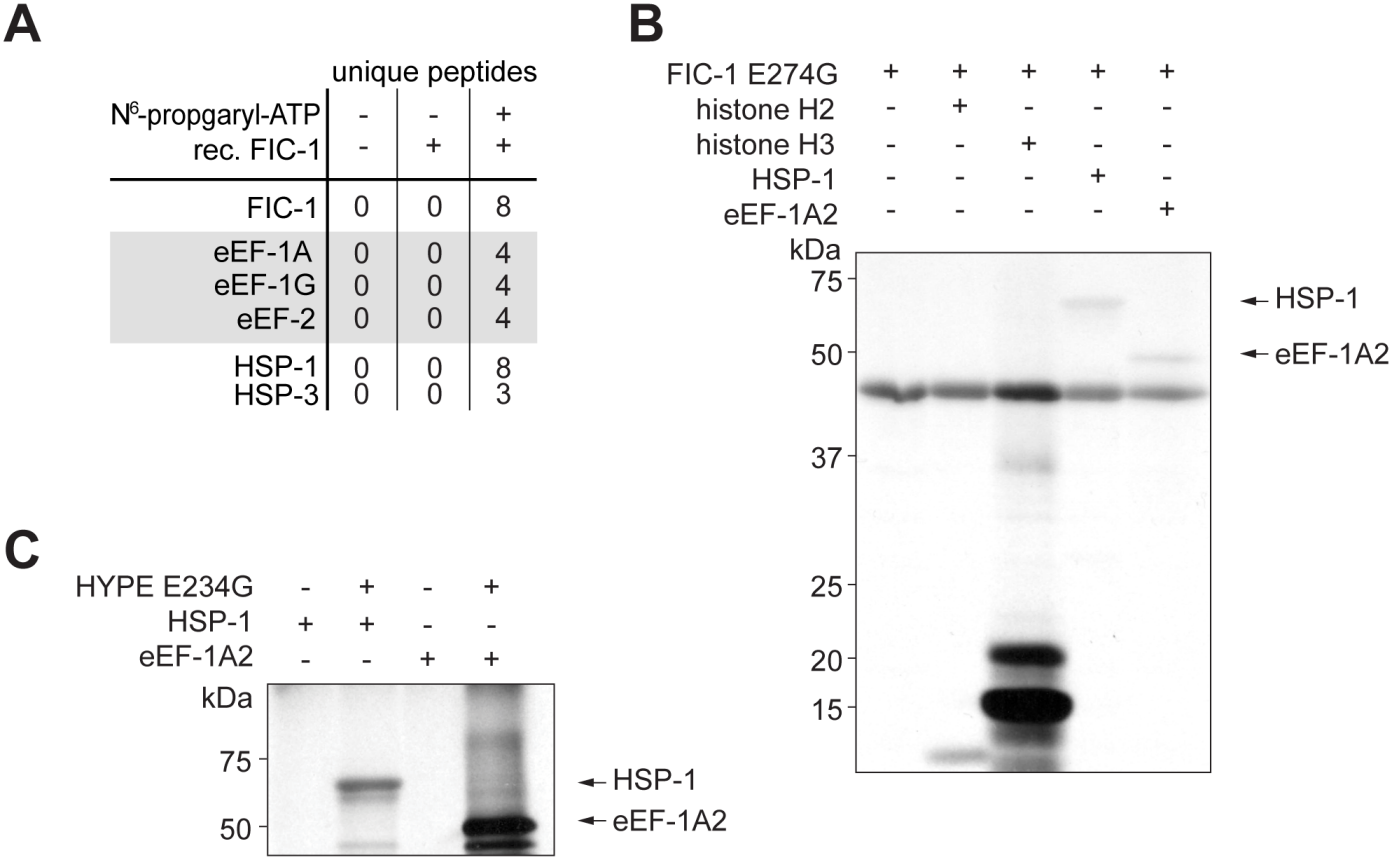
FIC-1 AMPylates conserved heat shock 70 family proteins and translation elongation factors. (A) Identification of new FIC-1 targets by mass spectrometry. (B) Validation of novel FIC-1 targets: Recombinant FIC-1 E274G was incubated with α ^33^P-ATP for an hour at which point substrates (histone H3, HSP-1 or eEF-1A2) were added and the mixture was incubated for an additional hour. Sample autoradiography was assessed. (C) Novel FIC-1 targets are modified by HYPE: Recombinant HYPE E234G was incubated with α ^33^P-ATP for an hour at which point substrates (HSP-1 or eEF-1A) were added and the mixture was incubated for an additional hour. Sample autoradiography was assessed.

While we were able to demonstrate that FIC-1 AMPylates core histones *in vitro*, we hypothesized that there might be additional FIC-1 targets potentially linking AMPylation to innate immunity. To identify these proteins, we adapted a click-chemistry based approach, in which we spiked *C*. *elegans* total cell lysate with recombinant FIC-1 protein in the presence of N^6^-propargyl-ATP as nucleotide substrate (S6A Fig) [21]. Following AMPylation, we completed the reaction with biotin-(PEG)_3_-azide to covalently couple a biotin handle to the ATP-bound propargyl group. We recovered AMPylated and thus biotinylated proteins from total *C*. *elegans* lysate on Streptavidin-modified agarose beads. Bound proteins were eluted and analyzed by LC/MS/MS. A comparison of the hit list with two independent controls to eliminate false positives led to identification of two classes of proteins over-represented amongst the AMPylated fraction of proteins: HSP 70 proteins (HSP-1, HSP-3) as well as translation elongation factors (eEF-1A, eEF-1G, eEF-2) (Fig 6A). Heat-shock proteins possess chaperone activity: they bind to unfolded or misfolded targets and support their proper refolding or—when beyond repair—shuttle them towards degradation [22]. HSP-1 is predominantly cytosolic, whereas HSP-3 is retained within the ER lumen. Interestingly, HSP-3 is the *C*. *elegans* Bip/Grp78 ortholog. HSP-3—together with its close homolog, HSP-4—form a complex with IRE-1, ATF-6 and PEK-1, to preclude activation of UPR-related signaling events by preventing either oligomerization of IRE-1 and PEK-1 or proteolytic processing of ATF-6 in the golgi [23]. BiP/Grp78 and HSP-3 / HSP-4 share more than 70% sequence similarity, with most of the divergence localized to the very N and C termini (S6B Fig).

The second class of identified targets—translation elongation factors—regulate protein translation by coordinating the selection and binding of aminoacyl-tRNA to the ribosome’s A-site (eEF-1A / EF-Tu) and by controlling the translocation of the peptidyl-tRNA from the A-site to the P-site of the ribosome (eEF-2) [24, 25]. *C*. *elegans* eEF-1A and eEF-2 share more than 80% sequence similarity with their human orthologs (S6C Fig). To validate these targets, we recombinantly expressed and purified *C*. *elegans* representatives of each family, HSP-1, HSP-3 as well as eEF-1A2. We tested them for modification in an *in vitro* AMPylation reaction, which confirmed HSP-1, HSP-3 and eEF-1A2 as substrates for FIC-1 E274G (Fig 6B). The human ortholog HYPE E234G modified these new FIC-1 targets as well (Fig 6C), indicative of their functional similarities. Our results thus reveal new Fic protein targets and suggest a role for AMPylation in the regulation of the HSP-3-dependent branch of the UPR as well as protein translation, a combination of which might account for the observed changes in pathogen susceptibility.

### AMPylation site mapping on *C*. *elegans* proteins highlights poly-modifications

**Fig 7:**
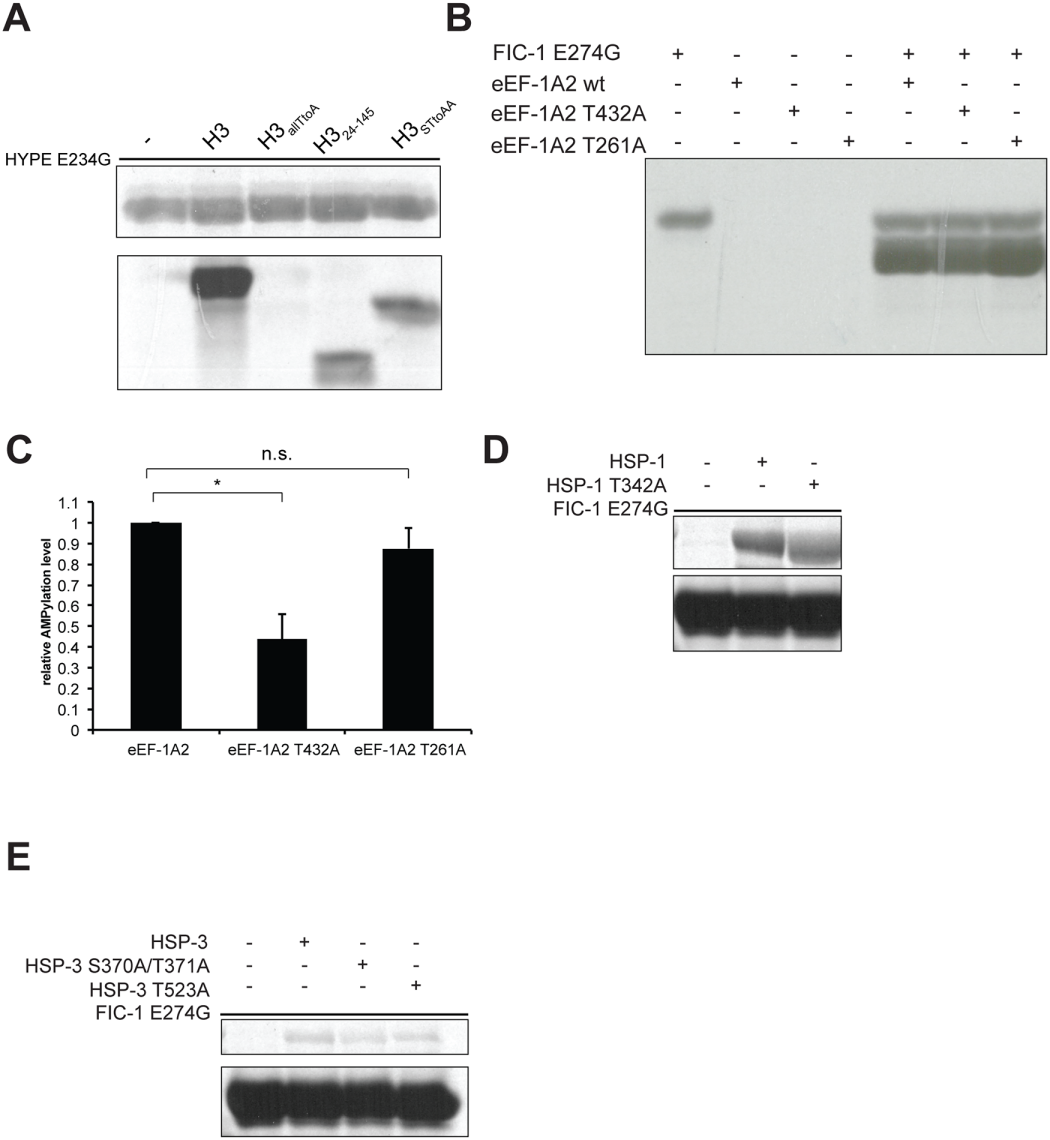
FIC-1 and HYPE AMPylate *C*. *elegans* targets on multiple sites. (A) HYPE AMPylates threonines on histone H3: Recombinant HYPE E234G was incubated with α ^33^P-ATP for an hour at which point substrates (histone H3 wild type and mutants) were added and the mixture was incubated for an additional hour. Sample autoradiography was assessed. (B-C) FIC-1 modifies eEF-1A2 on T432: Recombinant FIC-1 E274G was incubated with α ^33^P-ATP for an hour at which point substrates (eEF-1A2_244-463_ wild type and mutants) were added and the mixture was incubated for an additional hour. Sample autoradiography was assessed qualitatively (B) and quantitatively (C); data shown here represents the average of two independent replicas. (D-E) FIC-1 modifies HSP-1 and HSP-3 on distinct sites from human BiP: Recombinant FIC-1 E274G was incubated with α ^33^P-ATP for an hour at which point substrates (HSP-1, HSP-3 and respective mutants) were added and the mixture was incubated for an additional hour. Sample autoradiography was assessed.

AMPylation by eukaryotic FIC proteins is a site-specific process where threonines represent the preferred sites of modification. To characterize AMPylation of *C*. *elegans* targets by FIC-1 E274G or HYPE E234G, we used a combined approach of LC-MS/MS analysis and *in vitro* AMPylation assays, testing recombinantly expressed targets with specific mutations that alter presumptive sites of AMPylation. We previously showed that histone H3 is not modified on tyrosines by HYPE E234G [13]. As eukaryotic FIC proteins preferentially modify threonines, we constructed, purified and tested three additional histone H3 mutants: histone H3_allTtoA_ has all threonines replaced with alanines, H3_24-145_ misses the N-terminal 24 amino acids among which are four threonines and H3_STtoAA_ has two serine/threonine (S/T) motifs mutated to alanine/alanine (A/A). We observed that HYPE E234G was unable to AMPylate H3_allTtoA_ while overall AMPylation levels of H3_24-145_, and H3_STtoAA_ were decreased, as compared to wild type H3 (Fig 7A). In contrast, FIC-1 E274G did not modify any of these histone H3 mutant proteins (S7A Fig). To address eEF-1A2 AMPylation, we first purified a truncated eEF-1A2 version (eEF-1A2_244-463_), modified it in *in vitro* AMPylation reactions with FIC-1 E274G and analyzed the sample by LC-MS/MS. We detected two AMPylation sites, T269 and T432, with high confidence. Since AMPylation of human eEF-1A2 was recently mapped to T261, we recombinantly purified eEF-1A2_244-463_ T261A as well as eEF-1A2_244-463_ T432A, and tested these proteins in *in vitro* AMPylation assays using α-P^33^-ATP as nucleotide source. In reactions with FIC-1 E274G, we observed a significant reduction in eEF-1A2_244-463_ T432A AMPylation while eEF-1A2_244-463_ T261A modification was indistinguishable from EEF-1A2_244-463_ wild type (Fig 7B and 7C). We did not observe a significant difference in AMPylation levels when using HYPE E234G as AMPylator in reactions with EEF-1A2_244-463_ constructs (S7B Fig). To map the sites of AMPylation on HSP-1 and HSP-3, we first subjected *in vitro* modified proteins to LC-MS/MS analysis and identified T176 on HSP-3 as the only site of modification. The human HSP-3 orthologue BiP was previously shown to be modified on T366 and T518, respectively [14, 16]. This prompted us to clone and purify HSP-1 and HSP-3 versions with mutations in the respective BiP S365/T366 or T518 orthologue residues (HSP-1 T342A, HSP-3 S370A/T371A, HSP-3 T523A). We first tested these mutant HSP-1 and HSP-3 versions in *in vitro* AMPylation assays using FIC-1 E274G as AMPylator and observed no significant changes in AMPylation levels as compared to wild type HSP-1 or HSP-3 (Fig 7D and 7E). Contrasting, HYPE E234G-medatied AMPylation of HSP-1 T342A was reduced as compared to wild type HSP-1 while HSP-3 AMPylation levels didn’t fluctuate (S7C and S7D Fig). Together, our results highlight that *C*. *elegans* proteins are AMPylated on multiple sites, which do not necessarily overlap with modified human orthologues.

## Discussion

Fic protein-mediated target AMPylation is a conserved post-translational modification that may serve to regulate target activity. The ER-resident *D*. *melanogaster* Fic protein, dFic, as well as its human ortholog, HYPE, AMPylate BiP, an ER chaperone involved in the regulation of the UPR [15, 16]. Further, HYPE also modifies core histones H2-H4 *in vitro*, suggesting a possible role for HYPE in stress and DNA damage control [13, 14]. However, the intracellular localization of eukaryotic Fic proteins, their regulation, the identity of the physiologically most relevant targets, as well as their role in modulating cellular signaling events remain elusive.

Here we biochemically characterized the *C*. *elegans* Fic protein FIC-1, solved its structure and investigated its role in *C*. *elegans* stress tolerance *in vivo*. As expected based on the level of conservation of the catalytic and regulatory domains, FIC-1 acts as an AMPylase, capable to add an AMP entity to itself (auto-AMPylation) or to a target protein (target AMPylation). We map the auto-AMPylation sites to T352 and T476, two surface-exposed amino acids that are far from the active site of either monomer in the dimeric structure. T352 sits on the highly flexible flap structure that is likely to be accessible for self-modification. However, auto-AMPylation of T476 is hard to rationalize with respect to its physical position relative to the active site. Thus, AMPylation of T476 might therefore represent trans-AMPylation events rather than self-modifications. Since we purified and tested FIC-1_134−508_ and FIC-1_258−508_ only, we were not able to confirm any modifications on the orthologues of mapped HYPE self-modification sites T76, T80 or T183 [14]. However, HYPE_aa187-437_ E234G, which lacks all 3 presumable auto-AMPylation sites, remains fully active, displays self-modification and is capable of target modification.

Similar to HYPE and dFic, FIC-1 is hardly active in *in vitro* assays, while a single point mutation in the regulatory site (FIC-1 E274G) renders the enzyme more active without obviously altering its target specificity or increasing its promiscuity. We hypothesize that *in vivo*, FIC-1 is an efficient AMPylator, comparable to FIC-1 E274G, if a proper activation signal is provided, for example through interaction with (a) relevant partner protein(s). However, no such Fic protein regulators have been described yet.

We identify HSP-1, HSP-3 as well as eEF-1A, eEF-1G and eEF-2 as novel FIC-1 targets, all belonging to conserved protein families. HSP-1 and HSP-3 are heat-shock family 70 proteins and share >80% amino acid similarity with their human counterparts HSC-70 and BiP/Grp78, respectively. The human heat-shock 70 family protein BiP is AMPylated by HYPE on S365/T366 or T518 [15, 16]. Residues S365/T366 are highly conserved and present in BiP homologs found across species. *C*. *elegans* HSP-1, HSP-3 and HSP-4 all contain the very same amino acids as part of a strictly conserved amino acid stretch near these proteins’ C termini (LVGGS**T**RIPK). In contrast, T518 of human BiP is present only in HSP-3 and absent from HSP-1 and HSP-4. We map HSP-3 AMPylation to T176, yet another amino acid that is part of a strictly conserved sequence motif shared among BiP homologs (AVV**T**VPAYFND). We further confirm that neither of the orthologous BiP AMPylation sites, S365/T366 and T518, are modified on HSP-1 or HSP-3 by FIC-1. Modification of different sites on orthologous proteins might reflect minor selectivity distinctions between HYPE and FIC-1. Notably, we observe that HYPE E274G preferentially modifies T342 on HSP-3, the equivalent to T366 in BiP. Whether AMPylation of different—or even multiple—sites on Heat shock 70 family proteins results in diverse changes of their activities remains to be studied.

The regulation of the HSP-3 / HSP-4 ortholog BiP by post-translational modifications is not a new concept: first, BiP is modified by ADP-ribosyltransferases on R470 and 492 in the substrate binding domain, resulting in reversible BIP inactivation [26]. Second, BiP is auto-phosphorylated on a threonine residue in close proximity to the catalytic cleft, presumably as a consequence of BiP’s inherent ATPase activity [27]. The consequences of BIP AMPylation are a matter of debate. Cells that experience ER stress up-regulate HYPE, presumably increasing AMPylation of BIP and other target proteins. BIP AMPylation was hypothesized to facilitate its dissociation from a set of substrates that include IRE-1, PEK-1 and ATF-6 to support the initiation of the UPR. In this study, we explored a possible link between AMPylation and the induction of an ER stress response, but we failed to observe a strong connection between AMPylation activity and the UPR, nor did we see connections between AMPylation activity and early development or survival under conditions of ER stress. Our findings that AMPylation had no measurable effect on the IRE-1 / XBP-1 branch of the UPR are in accordance with published results, demonstrating that under acute ER stress conditions, HYPE-mediated target AMPylation is required for the activation of PERK and ATF6-based UPR cascades but not for IRE-1 activation [14]. Thus, the inbuilt redundancy in the regulation of the UPR may mask the consequence of Hypo- and Hyper-AMPylation on a organismic level. While we hypothesize that AMPylation of HSP-3 and other factors could affect ER signaling, we conclude from our data that FIC-1 does not obviously regulate the UPR in *C*. *elegans* and may represent a “soft” rather than a major regulator of the UPR.

The cytosolic heat shock protein HSP-1 is involved in the regulation of nuclear export of DAF-16 following physiological stress. Similar to DAF-16, HSP-1 is exclusively cytosolic but upon stress partially relocalizes to the nucleus [28].

Like heat shock proteins, the identified translation elongation factors eEF-1A, eEF-1G and eEF-2 are > 75% identical to their human orthologs. Modifications of translation elongation factors by Fic proteins also occur in bacteria. The P1 bacteriophage-encoded Fic protein Doc phosphorylates *E*. *coli* EF-Tu at position T382, inhibiting translation [8, 9]. AMPylation of elongation factors may therefore alter translation in affected cells. We mapped eEF-1A2 poly-AMPylation by FIC-1 to T269 and T432. HYPE modifies human eEF-1A on T261 [29]. We find that T261 is not altered by FIC-1 or HYPE in *in vitro* reactions using *C*. *elegans* eEF-1A2 as substrate. *C*. *elegans* T432 is the residue that corresponds to *E*.*coli* elF-Tu T382, indicating overlapping target site preferences between *E*.*coli* Doc and FIC-1. Of particular interest is the fact that eEF-1A2 is modified on two distinct sites. Whether or not these sites are simultaneously engaged, resulting in target oligo-AMPylation, or modified independently remains to be investigated.

Finally, FIC-1 also reliably AMPylates core histones H2 and H3 at least *in vitro*. Histones are among the most conserved proteins found in nature, with the human and *C*. *elegans* versions sharing ~ 80% and up to 97% (H3) or 98% (H4) sequence identity [30]. HYPE AMPylates core histones H2-H4 but not H1 [13]. FIC-1 AMPylates histones H2-H3 but not H1 or H4. In contrast to AMPylation, histone methylation occurs on lysines or arginines contained in the N-terminal flexible histone tail, while histone acetylation is restricted to lysine residues [31, 32]. Histones may also be phosphorylated on serines, threonines and tyrosines [33], an important modification during the DNA damage response, when phosphorylated histone H2A assembles in chromatin domains at sites of DNA breakage. Further, Chk-1-mediated histone H3 phosphorylation on T11 rapidly decreases in cells experiencing DNA damage, resulting in the repression of a set of genes including Cdk1 and cyclin B1 [34]. Threonine residues are the preferred targets for HYPE and FIC-1. Removal of the N-terminal 24 amino acids or mutation of the two serine/threonine motifs in histone H3 greatly reduced HYPE-mediated and abolished FIC-1-mediated H3 AMPylation, strongly suggesting oligo-AMPylation by HYPE. However, we cannot exclude the possibility that these mutations distort the overall fold of histone H3, thus preventing AMPylator-H3 interactions and subsequent modifications. Threonine oligo-AMPylation may mimic histone phosphorylation and play a role in the DNA damage response, too.

Despite our best efforts, the ability to recover the modified endogenous targets in amounts that would enable an *in vivo* validation of our *in vitro* data has so far exceeded our experimental capabilities. Thus, it remains to be tested which proteins may represent the primary *in vivo* targets of FIC-1 in *C*. *elegans*.

To fully characterize the *C*. *elegans* protein FIC-1, we solved the atomic structure of FIC-1 and FIC-1 E274G. FIC-1 and HYPE are structurally very similar despite their sequence divergence (38% amino acid sequence identity), which may explain the partially overlapping specificity of these two enzymes. As observed for HYPE E234G, removal of the auto-inhibitory glutamate in FIC-1 did not cause obvious structural changes that could account for the massive increase in enzyme activity. While the TPR domain—a known protein-protein interaction module—is likely to bridge interactions of other proteins to FIC-1 and HYPE, we identified a second potential interaction site for binding partners opposite the ATP binding groove [35]. Given its location in close proximity to the active site, this particular site might be occupied by FIC-1 activators / inhibitors and thus fulfill an important role in enzyme regulation. The recruitment of different modulators to this potential interaction site may also explain the observed differences in target specificities of the two enzymes. Further, like HYPE, FIC-1 crystallized as a dimer; the high level of conservation at the dimerization interface among metazoan Fic proteins suggests that FIC-1 might preferentially exist as a dimer in intact cells, too. Recent work on bacterial FIC proteins showed the existence of an inhibitory tetrameric NmFic complex in solution [36]. We failed to obtain evidence for FIC-1 tetramerization in our experiments. However, the disruption of the dimerization interface dramatically decreases self- and target AMPylation of FIC-1 E274G, suggesting that dimerization might enhance its activity. Whether FIC-1 dimerization has consequences on target specificity remains to be investigated.

Complementary to our biochemical and structural investigation of FIC-1, we assayed its role in *C*. *elegans* physiology. We observed FIC-1 transcripts (mRNA) in all embryonic, larval and adult stages, suggesting a role for FIC-1 throughout development. The intracellular distribution of FIC-1 resembles the localization pattern of HYPE. We also detect a fraction of FIC-1 in the cytoplasm. We hypothesize that this cytoplasmic FIC-1 fraction modifies cytosolic target proteins such as HSP-1. Although the presence of HYPE in the cytoplasm of human cells has not been previously reported, at least one study reported HYPE-dependent AMPylation of cytosolic proteins, likewise arguing for the presence of HYPE in the cytoplasm, [29]. Of note, FIC-1-HA displays at a slightly lower apparent molecular weight in the nuclear and ER fractions than in the cytoplasmic fraction. These small differences might be attributable either to the prevailing self-AMPylation status of FIC-1 in the distinct cellular compartments or reflect other, as yet uncharacterized, regulatory modifications of FIC-1. Despite the presence of FIC-1 at early developmental stages, *fic-1* deficient animals develop normally and show no defects in brood size, egg viability or egg development even in the presence of acute or chronic ER stress. Instead, we observed a moderately increased susceptibility to lethal *P*. *aeruginosa* infections in *fic-1* deficient nematodes and, accordingly, slightly enhanced pathogen tolerance upon hyper-AMPylation as induced either by FIC-1 E274G or several extra copies of the *fic-1* wild type gene as present in our *fic-1*(n5823;nIs734) rescue line. Unexpectedly, *hsp-3* nematodes showed a hyper-sensitivity phenotype with regard to egg development on *P*. *aeruginosa* as a food source. These results support a model in which certain branches of UPR are essential for survival in the presence of bacterial pathogens. Innate immunity in *C*. *elegans* is controlled by DAF-2, PMK-1 and, to a lesser degree, HSF-1 [37]. The p38 mitogen-activated protein kinase PMK-1 pathway orchestrates the up-regulation of CUB-like proteins as well as C-type lectins in response to pathogen infections [37]. In contrast, HSF-1, a transcription factor activated by physiological stresses such as elevated temperatures, was proposed to act downstream of DAF-2 / DAF-16 and independently of PMK-1 [28]. Among the genes regulated by HSF-1 are molecular chaperones of the heat-shock protein family (HSPs) that may indirectly add to the innate immune response by supporting the refolding of unfolded proteins resulting from the release of ROS as a measure to fight the bacterial infection. Whether FIC-1 is involved in the regulation of DAF-2, HSF-1 or PMK-1 based immunity traits will require more extensive genetic and biochemical analysis.

Recent work highlighted a novel link between post-translational histone modifications and innate immunity in *C*. *elegans*: RNAi ablation of the H3K4 methyltransferase set-16/MLL decreased H3K4me3 levels at infection-associated gene promotors, leading to reduced transcription of these genes [38]. Further, global K14me1 levels as well as the intracellular distribution of K14me1-modified linker histone H1 (HIS-24) change in response to bacterial infections in *C*. *elegans*, suggesting an epigenetic component in the induction of an innate immune response [39]. In addition to a potential role in the regulation of DNA damage responses, histone AMPylation might alter the transcription of infection-related genes. We are currently exploring such possible connections.

**Fig 8:**
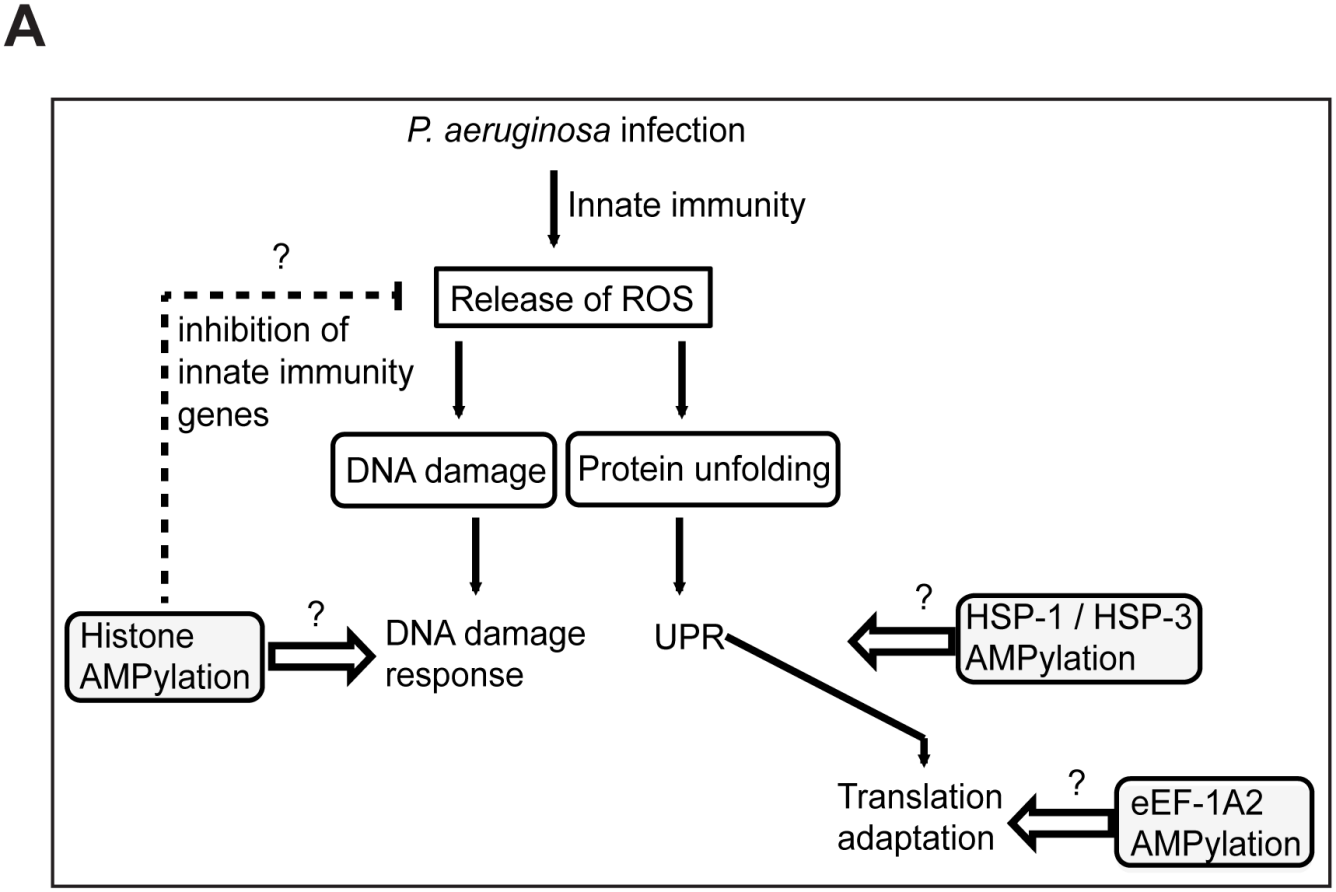
Proposed model. (A) Schematic representation of how FIC-1 might be involved in controlling antimicrobial responses in *C*. *elegans*.

We propose the following model to explain how FIC-1 mediated target AMPylation might alter protein functions that result in the phenotypes observed (Fig 8A): Infection of *C*. *elegans* with *P*. *aeruginosa* triggers an innate immune response, resulting in the release of reactive oxygen species (ROS). These molecules damage both DNA and proteins and indirectly initiate cellular repair mechanisms [40, 41]. In wild type animals exposed to stress, FIC-1 AMPylates HSP-1, HSP-3 and presumably HSP-4, thereby increasing dissociation of these chaperones from their intrinsic binding partner(s) and supporting the activation of the UPR in the ER lumen. Similar to phosphorylation, AMPylation of elongation factors could further contribute to UPR activation by inhibiting eEF-2, attenuating translation and increasing the specific expression of ATF-4, a transcription factor positively controlling the transcription of UPR-linked genes [42]. Histone AMPylation might limit the transcription of infection-associated genes and—together or in parallel with phosphorylation—trigger and support the repair of DNA damage introduced by ROS. Consequently, absence of AMPylation activity would result in slower induction of, or a less pronounced UPR and less efficient DNA damage repair. Vice versa, hyper-AMPylation could place cells in a primed state, able to immediately deal with any newly imposed stress. The observed changes in pathogen susceptibility, although reproducible and statistically significant, are minor, may be indirect and may not reflect the major cellular process(es) regulated by AMPylation. Thus we propose that FIC-1 should be seen as a soft modulator, rather than a master regulator of the signaling processes discussed above.

In this study, we have described how FIC-1 AMPylates heat shock 70 family proteins, histones H2 and H3, as well as translation elongation factors and provided evidence for a link between FIC-1 mediated target AMPylation and innate immunity in *C*. *elegans*. Whether FIC-1 mediates target AMPylation modulates antimicrobial defense mechanisms and contributes to DNA and protein damage repair will require more extensive genetic and biochemical analysis.

## Materials and Methods

*C*. *elegans* worms were maintained at 20°C on nematode growth medium (NGM) agar plates seeded with OP50 *E*.*coli* bacteria unless stated otherwise [43]. The following strains, mutations, integrations and extra-chromosomal arrays were used in this study:

SJ4005 (*zcIs4*[P*hsp-4*::*gfp*] V), RB1104 (*hsp-3*(*ok1083*) X), MT22519 (*nEx2219*[P*fic-1*::*GFP*, P*lin-44*::*GFP])*, MT22798 (*nEX2237*[P*fic-1*::FIC-1 E274G, P*myo-3*::mCherry]), MT22849 (*n5823* IV [*fic-1* KO]), MT23529 (*n5823* IV; *nIs734*), MT23188 (*n5823*; *nEx2318*), MT23262 (*n5823* IV; muIs109), MT23307 (*n5823* IV; *zcIs4* V), MT23265 (*n5823* IV; *zcIs13* V), MT23494 (*n5823* IV; *hsp-3*(*ok1083*) X), MT23495 (*hsp-3*(*ok1083* X; *nEx2237*)), MT23497 (*xbp-1*(*tm2482*) III; *n5823* IV), MT23498 (*xbp-1*(*tm2482*) III; *nEx2237*), MT23503 (*nIs733* [P*fic-1*::FIC-1 E274G, P*myo-3*::mCherry]), MT23506 (*zcIs4* V; *nIs733*), MT23508 (*zcIs13* V; *nIs733*), MT23527 (*daf-2*(*e1370ts*) III; *n5823* IV), MT23528 (*daf-2*(*e1370ts*) III; nIs733), MT23530 (*nEx2396*[P*hsp16*.*2*::*fic-1*; P*myo-3*::*mCherry*]), CB1370 (*daf-2*(*e1370ts*)) III, ZD418(*xbp-1*(*tm2482*) III, RB1104 (*hsp-3*(*ok1083*)), SJ4005 (*zcIs4* V [P*hsp-4*::GFP; lin-15(n765)]), SJ4100 (*zcIs13* V [P*hsp-6*::GFP])

### Plasmid construction

P*fic-1*::FIC-1 E274G was built in a two step cloning process. First, a 1.6 kBps PCR fragment spanning the putative *fic-1* promoter was used to replace the eat-4 promoter sequence in pNB7. Next, the *fic-1* gene was amplified from *C*. *elegans* total cDNA and cloned into the new vector. Point mutations in *fic-1* were introduced by SOEing PCR with the mutations encoded in the respective primers. The promotor trap construct (P*fic-1*::GFP) was cloned by replacing the *fic-1* gene with a *gfp* orf. For inducible protein expression, the *fic-1* gene was cloned into pPD49.78 (Fire lab vector kit) with an additional C-terminal HA-tag introduced as part of the primer Recombinant FIC-1 protein purification for *in vitro* AMPylation assays was based on *fic-1*_258−508_ cloned into a single orf of a pET-DUET1 vector. Primer sequences are available upon request. For *fic-1*(n5823) rescue, a 4.5 kbps linear DNA fragment was amplified using genomic DNA as template.

For crystallization, *fic-1*_134−508_ was cloned with a non-cleavable N-terminal 6x His tag into an Ampicillin resistant T7 based bacterial expression plasmid. The transmembrane helix and membrane proximal domain of FIC-1 were excluded from both the wild type and the E274G construct.

### CRISPR-mediated genome editing

CRISPR-mediated genome editing was performed essentially as previously described [44, 45]. In brief, day-1 adult animals were injected with P*eft-3*::*Cas9*::tbb-2–3′ UTR [44] (20 ng/μl), P*myo*-*3*::*mCherry* (10 ng/μl), P*rab-3*::*mCherry* (10 ng/μl), a 800 bps linear PCR fragment amplified from the U6-target-sgRNA scaffold plasmid [45] (50 ng/μl), pcDNA3.1 (100 ng/μl), in 1x TE buffer and grown at 20°C. 3 days after injection, mCherry-positive animals were separated and their F2 generation tested for homozygous *fic-1* modifications by sequencing. Lines of interest were back-crossed to wild type at least twice prior to use. MT22849 and derivate lines were routinely genotyped by PCR using 3 primers at once resulting in a 750 bps (wild type) or 950 bps (KO) product (S1C and S1D Fig).

### Germline transformation

Germline transformation was performed as described. The *gfp* reporter transgene (P*fic-1*::*GFP*) was injected at 50 ng/μl into *lin-15(n765ts)* animals with 50 ng/μl of pL15EK as a co-injection marker [46]. The rescue construct (4.5 kBps PCR fragment) was injected at 50 ng/μl into MT22433 with 10 ng/μl each of P*myo-3*::*mCherry* and P*rab-3*::*mCherry* as a co-injection markers. P*hsp-16*::*fic-1* was injected at 50 ng/μl into MT22433 with 10 ng/μl each of P*myo-3*::*mCherry* and P*rab-3*::*mCherry* as a co-injection markers.

### Small molecular fluorescence in situ hybridization (smFISH)

Fluorescence *in situ* hybridization was performed as described [18]. The *fic-1* smFISH probes (Biosearch Technologies, Inc) were conjugated to Cy5 fluorophores using the Amersham Cy5 Mono-reactive Dye pack (GE Healthcare). Probe sequences are available upon request. Images in Fig 4 are maximum intensity projections of Z-stacks processed with the FFT Bandpass Filter operations in the image processing program Fiji [47].

### Immunoblotting

Nematodes were washed off NGM plates, snap-frozen in liquid nitrogen and resuspended in 50 mM Tris/HCl pH 8.0, 150 mM NaCl, 5 mM EDTA, 1% NP-40, 0.1% SDS supplemented with protease inhibitor mix (Roche). Animals were further cracked by sonication (15 pulses, 30% maximal power), rested on ice for 30 minutes, centrifuged for 10 minutes at maximal speed, supplemented with SDS running buffer and subjected to SDS-PAGE. Proteins were transferred to a polyvinylidene difluoride (PVDF) membrane and probed with appropriate antibodies or sera; S1 Table lists all antibodies used in this study. Chemiluminescent signal was detected using a Western Lightning ECL detection kit (Perkin Elmer Life Sciences) and exposure to XAR-5 films (Kodak).

### Immunofluorescence staining

Adult nematodes were heat-shocked for 2 hours at 34°C and thereafter treated with hypochlorite solution. Eggs were processed as described in [48]. All images were collected on a PerkinElmer Ultraview Multispectral Spinning Disk Confocal Microscope equipped with a Zeiss 1.4 NA oil immersion 63x objective lens and a Prior piezo-electric objective focusing device for maintaining focus. Images were acquired with a Hamamatsu ORCA ER-cooled CCD camera controlled with Metamorph software. Post-acquisition image manipulations were made using Fiji software [47].

### Generation of FIC-1-specific mouse serum

BL52/B6 mice were subcutaneously primed with 100 μg FIC-1 in PBS supplemented with complete Freund’s adjuvant, boosted by intraperiotenal injection 4 weeks later with 20 μg in PBS and terminally bled 5 days later.

### Reporter assays

ER as well as mitochondria stress reporter tests were performed on NGM plates supplemented with 10 μg/ml tunicamycin and 35 μg/ml ethidium bromide, respectively, and scored after 24 hours (ER stress) or 72 hours (mitochondrial stress).

### Survival/development assays

For tunicamycin assays, regular NGM plates were supplemented with various amounts of tunicamycin (EMD Milipore) in DMSO at a concentration of 1 mg/ml. Total DMSO volumes were adjusted to exclude solvent effects on worm development. Tunicamycin plates were incubated at 20°C for 24 hours prior to use. *Pseudomonas aeruginosa* PA14 was grown on SKA plates as described [49]. In brief, *P*. *aeruginosa* was grown overnight in LB at 37°C with shaking. The following day, 7 μl of *P*. *aeruginosa* culture were transferred to the center of 3 cm SKA plates, kept at room temperature for 3 hours and subsequently incubated for 24 hours at 37°C and thereafter for 24 hours at 20°C. For development assays, eggs harvested from hypochlorite treatment of 1-day old adults were transferred onto assay plates and thereafter incubated at 25°C (*Pseudomonas* plates) or 20°C (tunicamycin assays) for 72 hours; (N > 200 eggs for each strain and treatment). Animal development was scored using the following scoring classes: older than L4, L3/L4, younger than L3. For survival assays, 30–40 L4 animals were picked onto 3–4 *Pseudomonas* plates each and subsequently incubated at 25°C. Worm survival was scored at least every 24 hours until the last animal had died. Animals were considered dead if repetitive (10x) poking with a platinum loop did not result in any visible body movement. Worms that died by exploding through the vulva or desiccating on the side of plates were censored. Data was processed using PRISM software and statistical significance was tested using a Gehan-Breslow-Wilcoxon test.

### Longevity assays

L4 animals were transferred onto fresh NGM plates and subsequently picked onto new NGM plates at two-day intervals for 14 days. Animals were considered dead if repetitive poking (10x) with a platinum loop did not result in any visible body movement. Worms that died by exploding through the vulva or desiccating on the side of plates were censored.

### Protein purification

Purification of recombinant HYPE_aa187-437_ E234G and FIC-1_258−508_ for *in vitro* AMPylation assays as well as HSP-1, HSP-1 T342A, HSP-1 T496A, HSP-3, HSP-3 S370A/T371A, HSP-3 T523A was performed following methods described in [13]. eEF-1A2, eEF-1A2_244-463_, eEF-1A2_244-463_ T261A eEF-1A2_244-463_ T432A as well as histone H3_allTtoA_, H3_24-145_ and H3_noSTmotif_ were purified under denaturing conditions as described in [13].

For crystallization, FIC-1_134−508_ was expressed in *E*. *coli* LOBSTR-RIL(DE3) (Kerafast) [50]. Transformed cells were grown at 37°C to an OD_600_ of 0.6, the temperature was shifted to 18°C and expression was induced by the addition of isopropyl β-D-1-thiogalactopyranoside to a final concentration of 0.2 mM for 16 hours. Cells were harvested by centrifugation at 6000 g, resuspended in lysis buffer (50 mM potassium phosphate, pH 8.0, 400 mM NaCl, 40 mM imidazole, 2 mM MgCl_2_, 5 mM DTT, and 1 mM PMSF) and lysed using a cell disruptor (Constant Systems). The lysate was cleared by centrifugation at 10000 g for 25 minutes. The soluble fraction was incubated with Ni-Sepharose 6 Fast Flow beads (GE Healthcare) for 30 min at 4°C. The beads were washed with lysis buffer, and the protein was eluted (250 mM imidazole, 20 mM Tris/HCl pH 8.0, 150 mM NaCl, 2 mM MgCl_2_ and 5 mM DTT) and concentrated for further purification. Samples were purified to homogeneity via size-exclusion chromatography on a Superdex S200 26/60 column (GE Healthcare) equilibrated in running buffer (20 mM Tris-HCl, pH 8.0, 150 mM NaCl, 2 mM MgCl_2_, and 5 mM DTT). Finally, proteins were concentrated to 30 mg/ml, flash frozen in liquid nitrogen and stored at -80°C until usage. FIC-1_134−508_ E274G/T476A, FIC-1_134−508_ E274G/T352A, FIC-1_134−508_ E274G/V292D, FIC-1_134−508_ E274G/I298D were purified accordingly.

### Crystallization

Both wild type FIC-1 and the E274G constructs crystallized in the same condition. The initial crystal hit was obtained at a concentration of 1.8 mg/ml in Protein Complex Suite (Qiagen) screen condition F9. Larger crystals were obtained via hanging drop vapor diffusion and grew over two weeks at 18°C in 0.1 M MES pH 6.5 and 1.1 M ammonium sulfate using a 1:1 drop ratio with mother liquor. Crystals were harvested and cryo-protected in mother liquor with 16% (v/v) glycerol, in the presence of 5 mM MgCl_2_ and 5 mM AMP-PNP; however, no ligand was detected in the structure.

### Data collection and structure determination

Data was collected at beamline 24 at the Advanced Photon Source at Argonne National Laboratories. All data processing was done using programs provided by SBgrid [51]. Data reduction was performed with HKL2000, molecular replacement was done with PHASER, using a monomer from the human Fic-domain containing protein, HYPE (PDB code 4U07) as a search model [52]. Two molecules were readily found in the asymmetric unit. The structures were manually built using Coot and refined with phenix.refine [53]. Data collection and refinement statistics are summarized in S2 Table.

### In vitro AMPylation assays

In vitro AMPylation were performed essentially as described in [13]. In brief, 5–10 μg of FIC-1 in 10 μl were first mixed with 25 μl of 50 mM Tris/HCl pH 7.5, 150 mM NaCl, 7.5 mM MgCl_2_ 2 mM DTT supplemented with 0.5 μCi αP^33^-ATP. The reaction was allowed to proceed for 1 hour at room temperature, followed by the addition of 1–5 μg target protein (histones, HSP-1, HSP-3, eEF-1A2) in a 10 μl volume. The reaction mixture was incubated for another hour at room temperature, supplemented with 10 μl reducing SDS-sample buffer, boiled for 5 minutes and analyzed on 10–15% SDS-polyacrylamide gels.

For AMPylation site mapping, analogue reactions were performed using cold ATP (1 mM final concentration) instead of 0.5 μCi αP^33^-ATP. Samples were reduced, alkylated and digested with trypsin at 37°C overnight. The resulting peptides were extracted, concentrated and injected onto a Waters NanoAcquity HPLC equipped with a self-packed Aeris 3 μ3 is equipped with a self-packed Ax 20 cm, Phenomenex). Peptides were eluted using standard reverse-phase gradients. The effluent from the column was analyzed using an Orbitrap Elite (ThermoFisher) mass spectrometer (nanospray configuration) operated in a data dependent manner. The resulting fragmentation spectra were correlated against custom databases with Mascot (Matrix Science) 2.5.1 and PEAKS (Bioinformatics Solutions) 7.5 [54] using an AMP adduct mass of 329.0525 Da.

## Supporting Information

S1 Fig Sequence analysis of CRISPR-mediated fic-1 KO line. (A) Schematic representation of CRISPR-induced deletion and frame shift in fic-1 KO animals. (B) Schematic representation of truncated FIC-1 protein as encoded by CRISPR-mediated FIC-1 KO animals. (C) Triple-Primer PCR based genotyping of fic-1 KO lines: individual animals were lysed in worm lysis buffer and analyzed by PCR using three primers. Homozygous fic-1 KO results in a single, heavier PCR fragment, homozygous wild type in a single, lighter PCR fragment and heterozygous animals present both a heavier and lighter PCR fragment. (D) Schematic representation of triple-primer PCR rational: major primer pair (fw1 and rv1) binds independently of CRISPR-induced deletion site while second forward primer (fw2) binds directly on site of deletion and can therefore only contribute to the PCR reaction if at least one wild type allele is present. PCR reaction is optimized to favor the production of the shorter fragment (fw2 and rv1) if all 3 primers would be able to bind simultaneously (wild type situation).
(TIF)

S2 Fig FIC-1 has no global impact on the onset of ER or mitochondrial stress responses. (A) FIC-1 does not control the onset of the ER stress response: zcIs4[*hsp-4*::GFP; lin-15(n765)], zcIs4;*fic-1*(n5823) and zcIs4;FIC-1[E274G](nIs733) animals were exposed to either tunicamycin or ethidium bromide. GFP-reporter expression was scored 24 hours post exposure scale bar equals 500 μm. (B) FIC-1 does not control the onset of the mitochondrial stress response: zcIs13[*hsp-6*::GFP], zcIs13;*fic-1*(n5823) and zcIs13;FIC-1[E274G](nIs733) animals were exposed to either tunicamycin or ethidium bromide. GFP-reporter expression was scored 72 hours post exposure scale bar equals 500 μm. (C) and (D) nematode development under acute ER stress: eggs of indicated lines were transferred to OP50 plates containing 5 μg/ml tunicamycin to induce acute ER stress. Embryo development was scored.
(TIF)

S3 Fig FIC-1 is enriched in the adult germline as well as nematode embryos and localizes to the nuclear membrane. (A) FIC-1 is enriched in nematode embryos: representative images of embryos containing a promotor-trap construct nEx2219[P*fic-1*::GFP]; scale bar equals 40 μm. (B) Assessment of inducible FIC-1 expression. nEx2396[P*hsp16*.*2*::*fic-1*; P*myo-3*::*mCherry*] animals were heat-shocked for 2 hours at 34°C and FIC-1 protein level was assessed by Western blotting using anti-HA antibodies. (C) Characterization of FIC-1-specific mouse serum: Indicated recombinant proteins were probed with FIC-1-specific serum. (D) FIC-1 E274G localizes to the nuclear envelope: Staining of uninduced (-Dox) and induced (+Dox) Hela cells inducibly expressing GFP-tagged FIC-1 E274G or GFP-tagged HYPE E234G and its localization analyzed by confocal microscopy; scale bar equals 10 μm.
(TIF)

S4 Fig Self-AMPylation mapping on FIC-1. (A) and (B) LC-MS/MS spectra depicting modified peptides (R)T(+329.05)TQVYVGR(F) (A) and (K)T(+329.05)SSDNILNSGDSK(L) (B). Representative spectral plots shown here.
(TIF)

S5 Fig Structural overlay of FIC-1 and HYPE. (A) and (B) Superposition of the FIC-1 dimer and the HYPE dimer; FIC-1 in yellow-maroon-orange, HYPE in pale blue silver. (C) Comparison of HYPE and FIC-1 active sites. Top panels show the fic core of FIC-1 in two different orientations, with conserved domains labeled. Bottom left panel displays a superposition of the fic cores of HYPE (grey) and FIC-1 (light orange), illustrating strong structural conservation. Bottom right panel shows hydrogen bonding between key residues of FIC-1 and ATP, with the residue number from HYPE shown in brackets.
(TIF)

S6 Fig Schematic representation of Click-chemistry based target identification. (A) Schematic representation of click-chemistry based target identification: recombinant FIC-1 was mixed with total *C*. *elegans* lysate in the presence of N_6_-propargyl-ATP and incubated at room temperature for 60 minutes. Thereafter, reaction was supplemented with a Azide-PEG_3_-Biotin linker and incubated for another hour. Targets were retrieved using strepdavidin beads and eluates were assessed by mass spectrometry. (B) HSP-3 and HSP-4 are very similar to human BiP: Comparison of amino acid sequence conservation of *C*. *elegans* HSP-3 and HSP-4 with its human orthologue BiP. (C) HSPs and eEFs are conserved proteins: Comparison of amino acid sequence conservation of *C*. *elegans* HSPs and eEFs with their respective orthologs in *D*. *melanogaster*, *M*. *musculus* and *H*. *sapiens*.
(TIF)

S7 Fig FIC-1 and Hype AMPylate *C*. *elegans* targets on multiple sites. (A) FIC-1 AMPylates threonines on Histone H3: Recombinant FIC-1 E274G was incubated with α ^33^P-ATP for an hour at which point substrates (Histone H3 wild-type and mutants) were added and the mixture was incubated for an additional hour. Sample autoradiography was assessed. (B) HYPE modifies eEF-1A2: Recombinant HYPE E234G was incubated with α ^33^P-ATP for an hour at which point substrates (eEF-1A2_244-463_ wild type and mutants) were added and the mixture was incubated for an additional hour. Sample autoradiography was assessed qualitatively. (C-D) HYPE modifies HSP-1 and HSP-3 on distinct sites from human BiP: Recombinant HYPE E234G was incubated with α ^33^P-ATP for an hour at which point substrates (HSP-1, HSP-3 and respective mutants) were added and the mixture was incubated for an additional hour. Sample autoradiography was assessed.
(TIF)

S8 Fig Additional longevity assays. (A)—(C) wild type, *fic-1*(*n5823)*, *fic-1*(n5823;nIs734) rescue and FIC-1[E274G](nIs733) animals were kept at either 20°C and survival was scored every other day. Depicted n refers to number of animals at experiment initiation; number in brackets represents total counted dead events. P-values (Gehan-Breslow-Wilcoxon test) as compared to N2 wild type control: (A) not significant; (B) not significant; (C) N2. vs *fic-1*(n5823, nIs734): 0.006; all others not significant.
(TIF)

S9 Fig Additional *Pseudomonas* killing assays. (A)—(G) wild type, *fic-1*(*n5823)*, *fic-1*(n5823;nIs734) rescue and FIC-1[E274G](nIs733) L4 animals of indicted lines were place in the center of a *P*. *aeruginosa* loan and nematode survival was scored once per day until last animal vanished. Depicted n refers to number of animals at experiment initiation; number in brackets represents total counted dead events. P-values (Gehan-Breslow-Wilcoxon test) are as compared to N2 wild type control.
(TIF)

S1 Table Antibodies used in this study. (PDF)

S2 Table Data collection and refinement statistics. (PDF)
