# Supplementary figures and images for: The Caenorhabditis elegans Protein FIC-1 Is an AMPylase That Covalently Modifies Heat-Shock 70 Family Proteins, Translation Elongation Factors and Histones
Source: PLoS Genet. 2016 May 3;12(5):e1006023. doi: 10.1371/journal.pgen.1006023 (PMC4854385; doi:10.1371/journal.pgen.1006023)

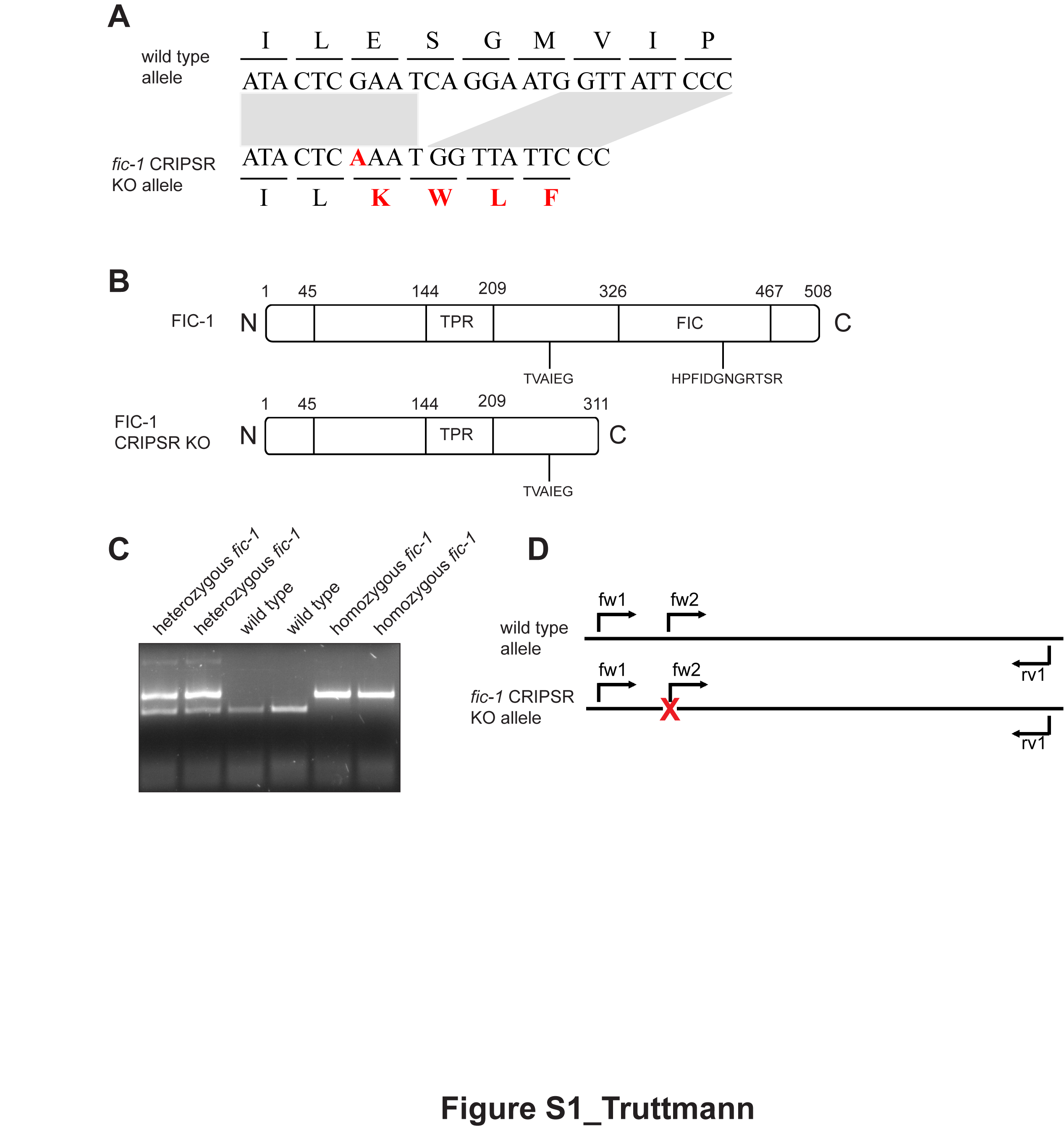

Supplement: S1 Fig — (A) Schematic representation of CRISPR-induced deletion and frame shift in fic-1 KO animals. (B) Schematic representation of truncated FIC-1 protein as encoded by CRISPR-mediated FIC-1 KO animals. (C) Triple-Primer PCR based genotyping of fic-1 KO lines: individual animals were lysed in worm lysis buffer and analyzed by PCR using three primers. Homozygous fic-1 KO results in a single, heavier PCR fragment, homozygous wild type in a single, lighter PCR fragment and heterozygous animals present both a heavier and lighter PCR fragment. (D) Schematic representation of triple-primer PCR rational: major primer pair (fw1 and rv1) binds independently of CRISPR-induced deletion site while second forward primer (fw2) binds directly on site of deletion and can therefore only contribute to the PCR reaction if at least one wild type allele is present. PCR reaction is optimized to favor the production of the shorter fragment (fw2 and rv1) if all 3 primers would be able to bind simultaneously (wild type situation). (TIF) [file pgen.1006023.s001.tif]

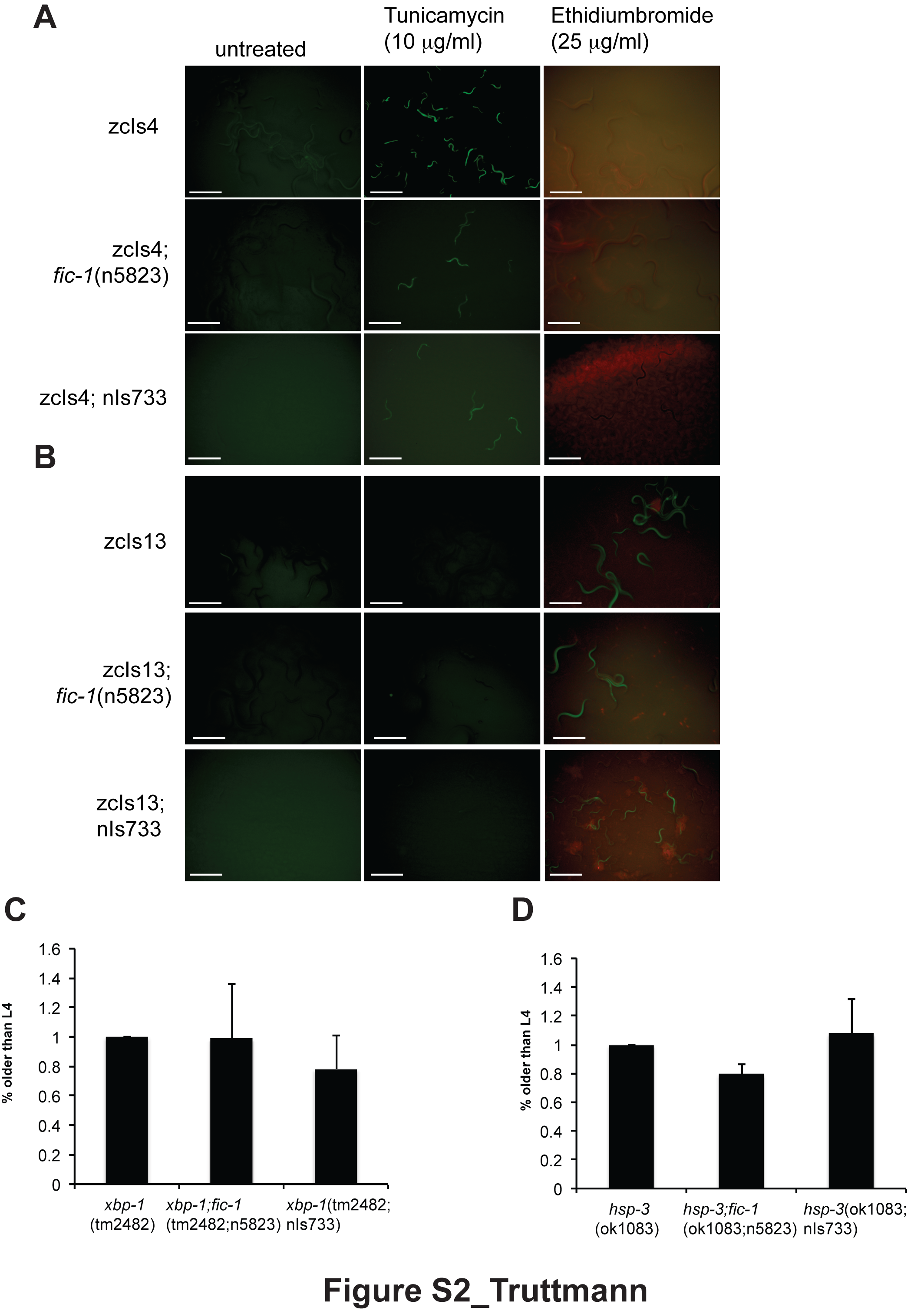

Supplement: S2 Fig — (A) FIC-1 does not control the onset of the ER stress response: zcIs4[hsp-4::GFP; lin-15(n765)], zcIs4;fic-1(n5823) and zcIs4;FIC-1[E274G](nIs733) animals were exposed to either tunicamycin or ethidium bromide. GFP-reporter expression was scored 24 hours post exposure scale bar equals 500 μm. (B) FIC-1 does not control the onset of the mitochondrial stress response: zcIs13[hsp-6::GFP], zcIs13;fic-1(n5823) and zcIs13;FIC-1[E274G](nIs733) animals were exposed to either tunicamycin or ethidium bromide. GFP-reporter expression was scored 72 hours post exposure scale bar equals 500 μm. (C) and (D) nematode development under acute ER stress: eggs of indicated lines were transferred to OP50 plates containing 5 μg/ml tunicamycin to induce acute ER stress. Embryo development was scored. (TIF) [file pgen.1006023.s002.tif]

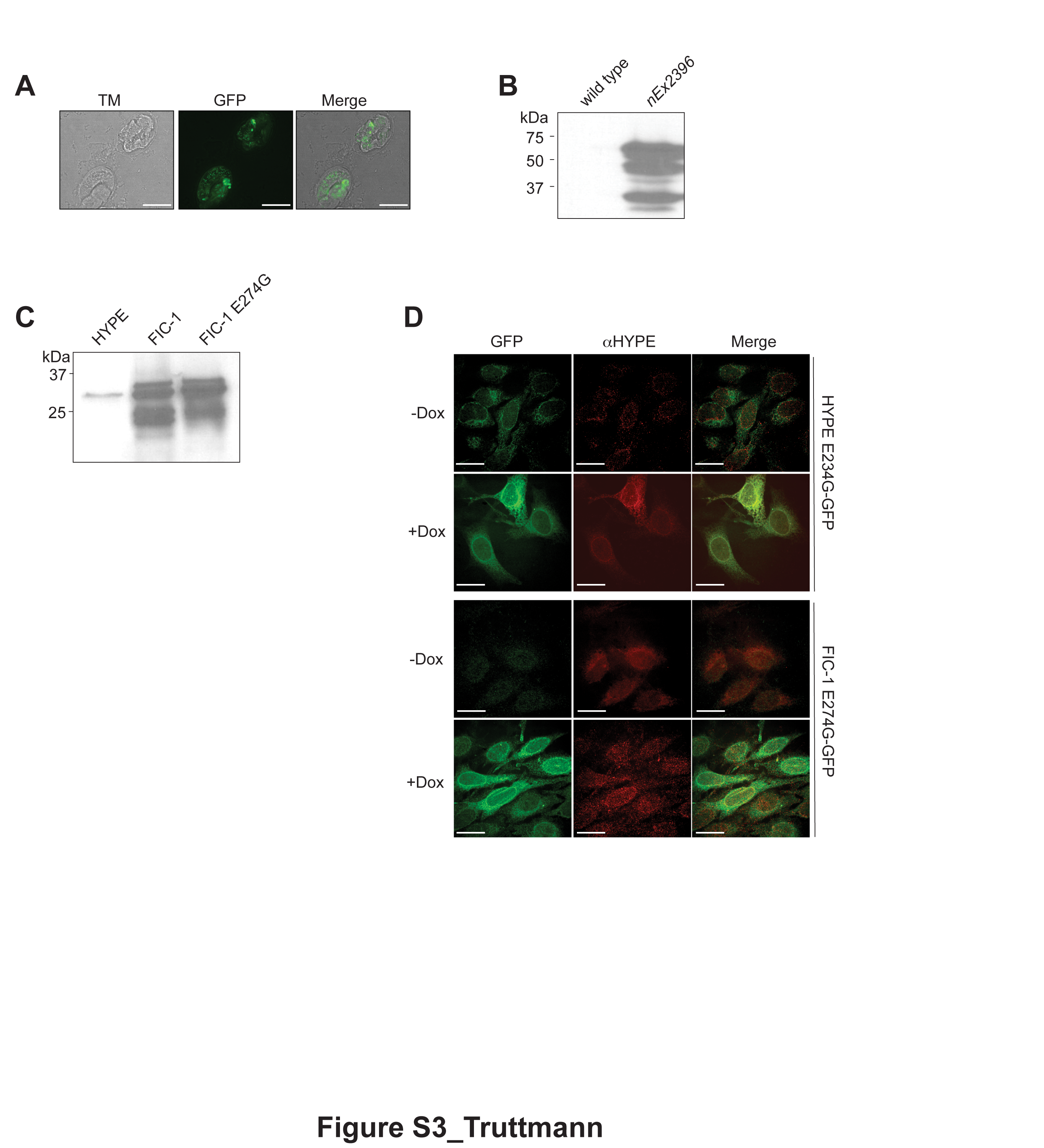

Supplement: S3 Fig — (A) FIC-1 is enriched in nematode embryos: representative images of embryos containing a promotor-trap construct nEx2219[Pfic-1::GFP]; scale bar equals 40 μm. (B) Assessment of inducible FIC-1 expression. nEx2396[Phsp16.2::fic-1; Pmyo-3::mCherry] animals were heat-shocked for 2 hours at 34°C and FIC-1 protein level was assessed by Western blotting using anti-HA antibodies. (C) Characterization of FIC-1-specific mouse serum: Indicated recombinant proteins were probed with FIC-1-specific serum. (D) FIC-1 E274G localizes to the nuclear envelope: Staining of uninduced (-Dox) and induced (+Dox) Hela cells inducibly expressing GFP-tagged FIC-1 E274G or GFP-tagged HYPE E234G and its localization analyzed by confocal microscopy; scale bar equals 10 μm. (TIF) [file pgen.1006023.s003.tif]

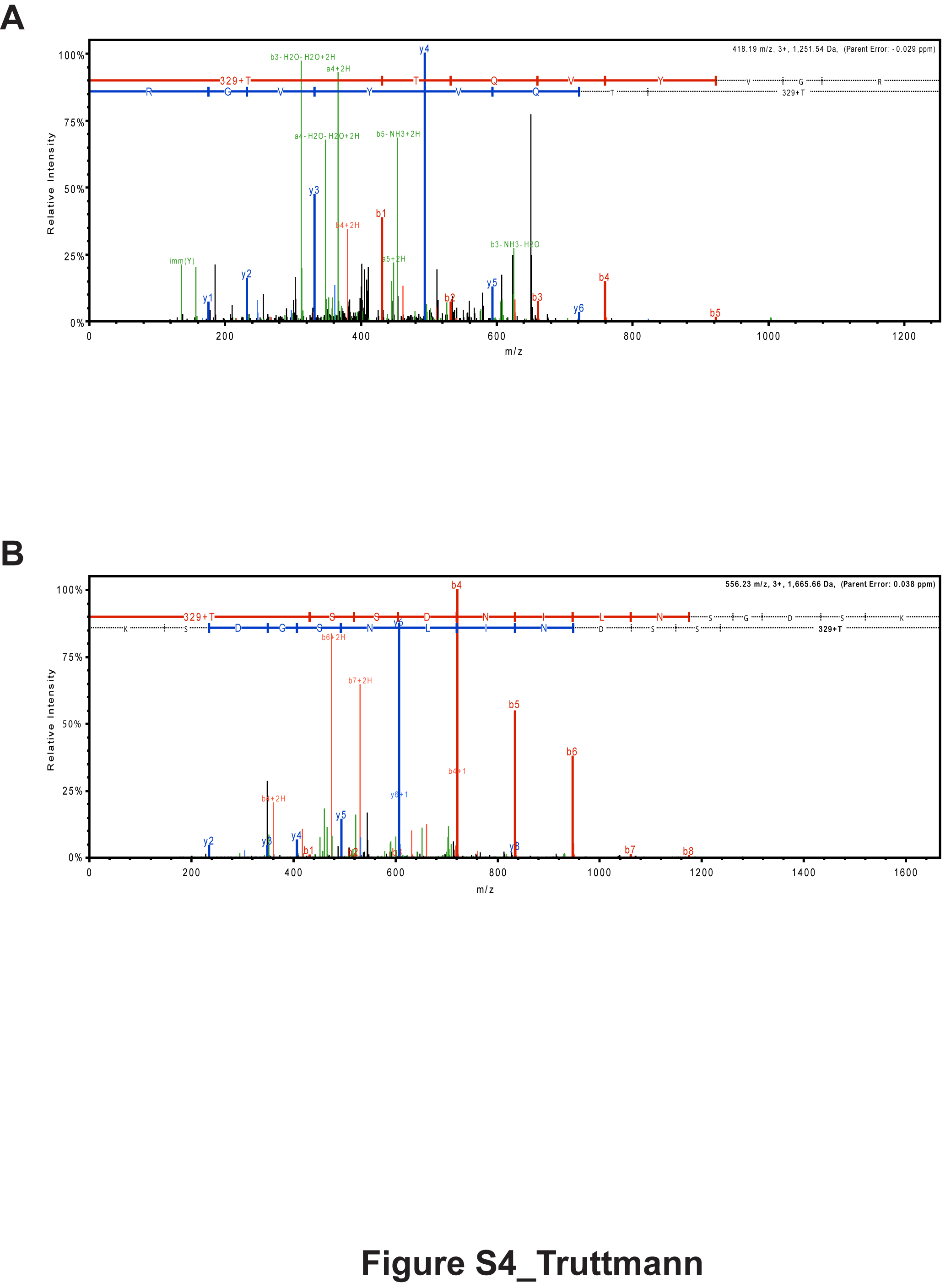

Supplement: S4 Fig — (A) and (B) LC-MS/MS spectra depicting modified peptides (R)T(+329.05)TQVYVGR(F) (A) and (K)T(+329.05)SSDNILNSGDSK(L) (B). Representative spectral plots shown here. (TIF) [file pgen.1006023.s004.tif]

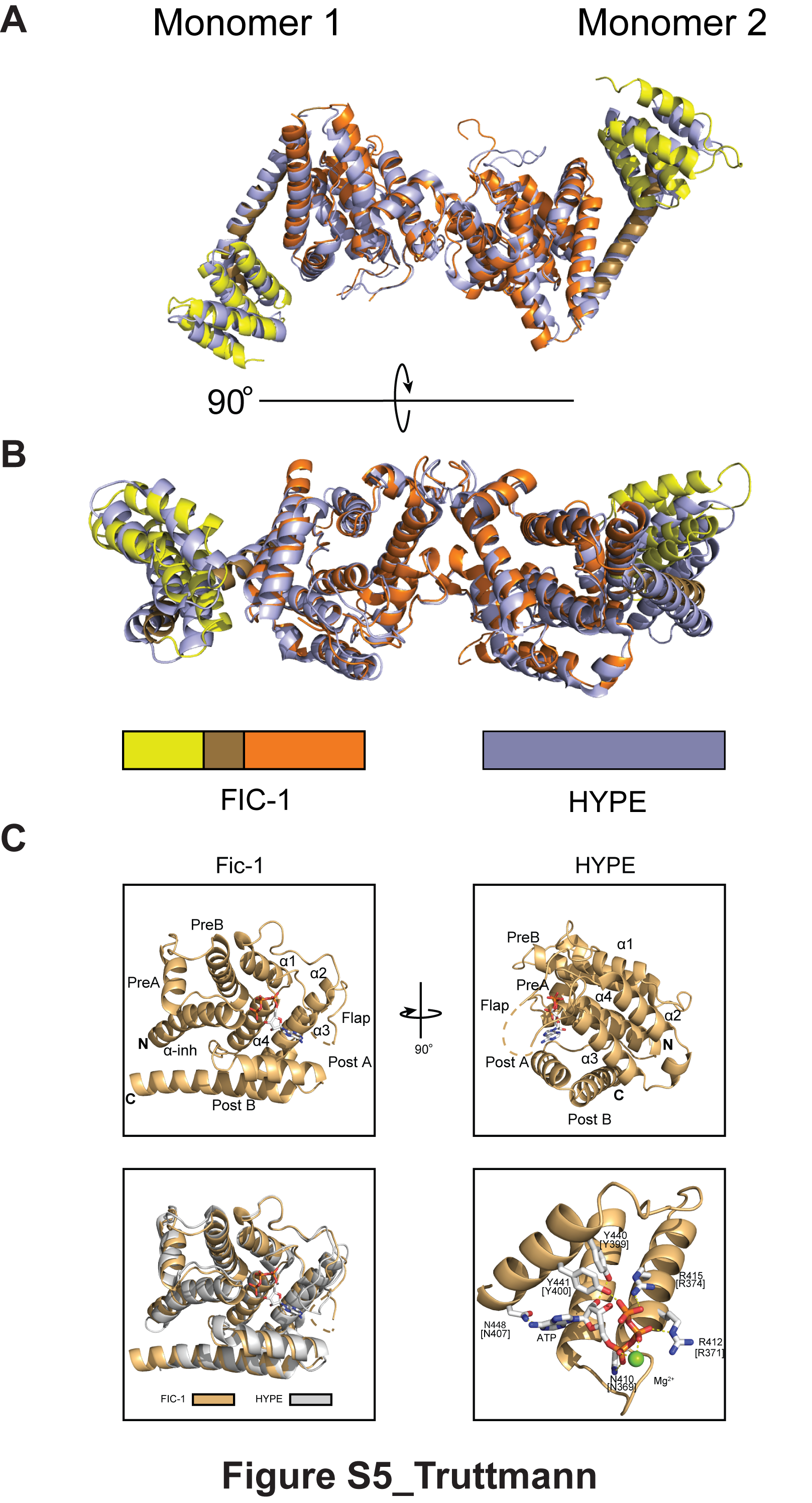

Supplement: S5 Fig — (A) and (B) Superposition of the FIC-1 dimer and the HYPE dimer; FIC-1 in yellow-maroon-orange, HYPE in pale blue silver. (C) Comparison of HYPE and FIC-1 active sites. Top panels show the fic core of FIC-1 in two different orientations, with conserved domains labeled. Bottom left panel displays a superposition of the fic cores of HYPE (grey) and FIC-1 (light orange), illustrating strong structural conservation. Bottom right panel shows hydrogen bonding between key residues of FIC-1 and ATP, with the residue number from HYPE shown in brackets. (TIF) [file pgen.1006023.s005.tif]

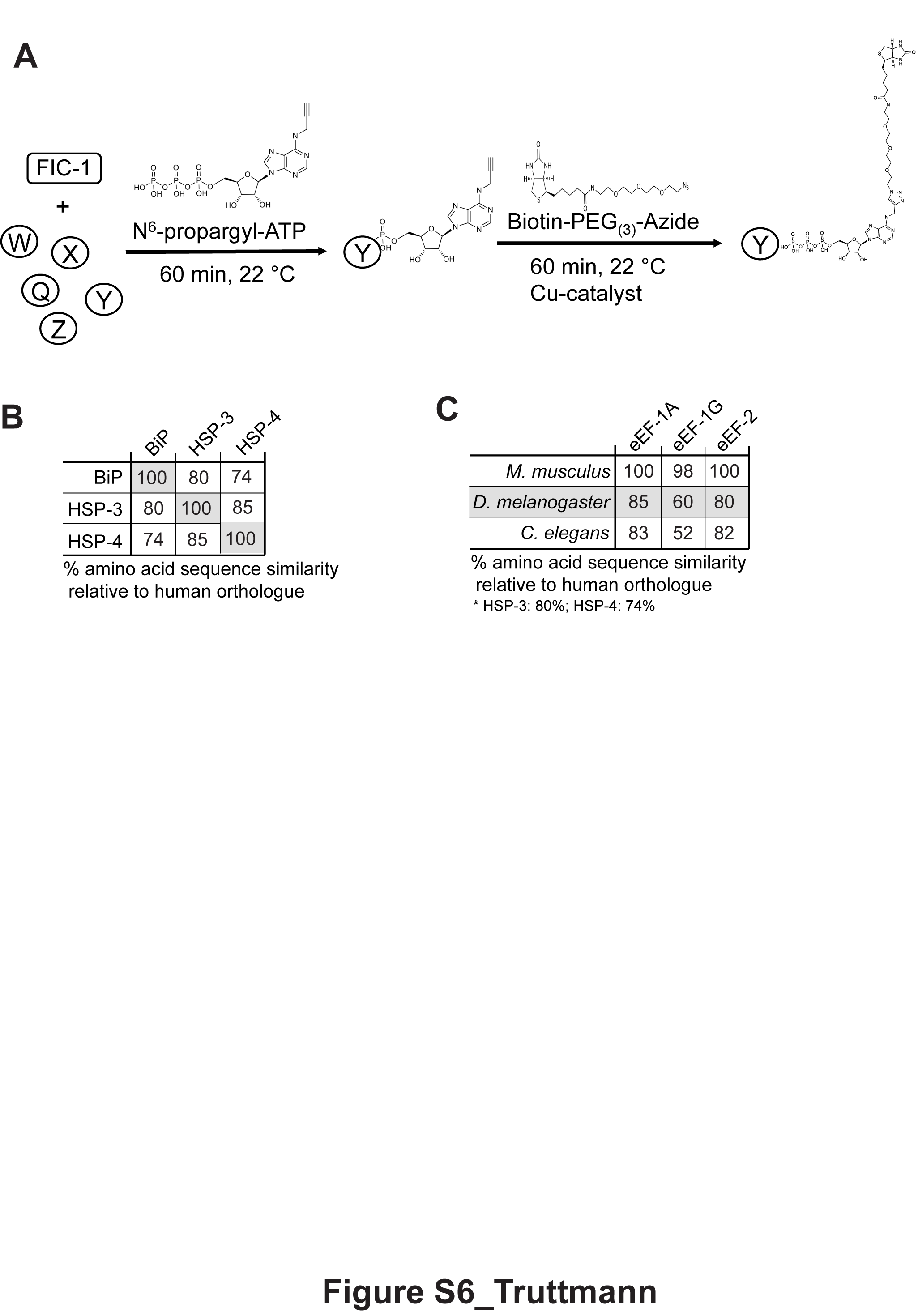

Supplement: S6 Fig — (A) Schematic representation of click-chemistry based target identification: recombinant FIC-1 was mixed with total C. elegans lysate in the presence of N6-propargyl-ATP and incubated at room temperature for 60 minutes. Thereafter, reaction was supplemented with a Azide-PEG3-Biotin linker and incubated for another hour. Targets were retrieved using strepdavidin beads and eluates were assessed by mass spectrometry. (B) HSP-3 and HSP-4 are very similar to human BiP: Comparison of amino acid sequence conservation of C. elegans HSP-3 and HSP-4 with its human orthologue BiP. (C) HSPs and eEFs are conserved proteins: Comparison of amino acid sequence conservation of C. elegans HSPs and eEFs with their respective orthologs in D. melanogaster, M. musculus and H. sapiens. (TIF) [file pgen.1006023.s006.tif]

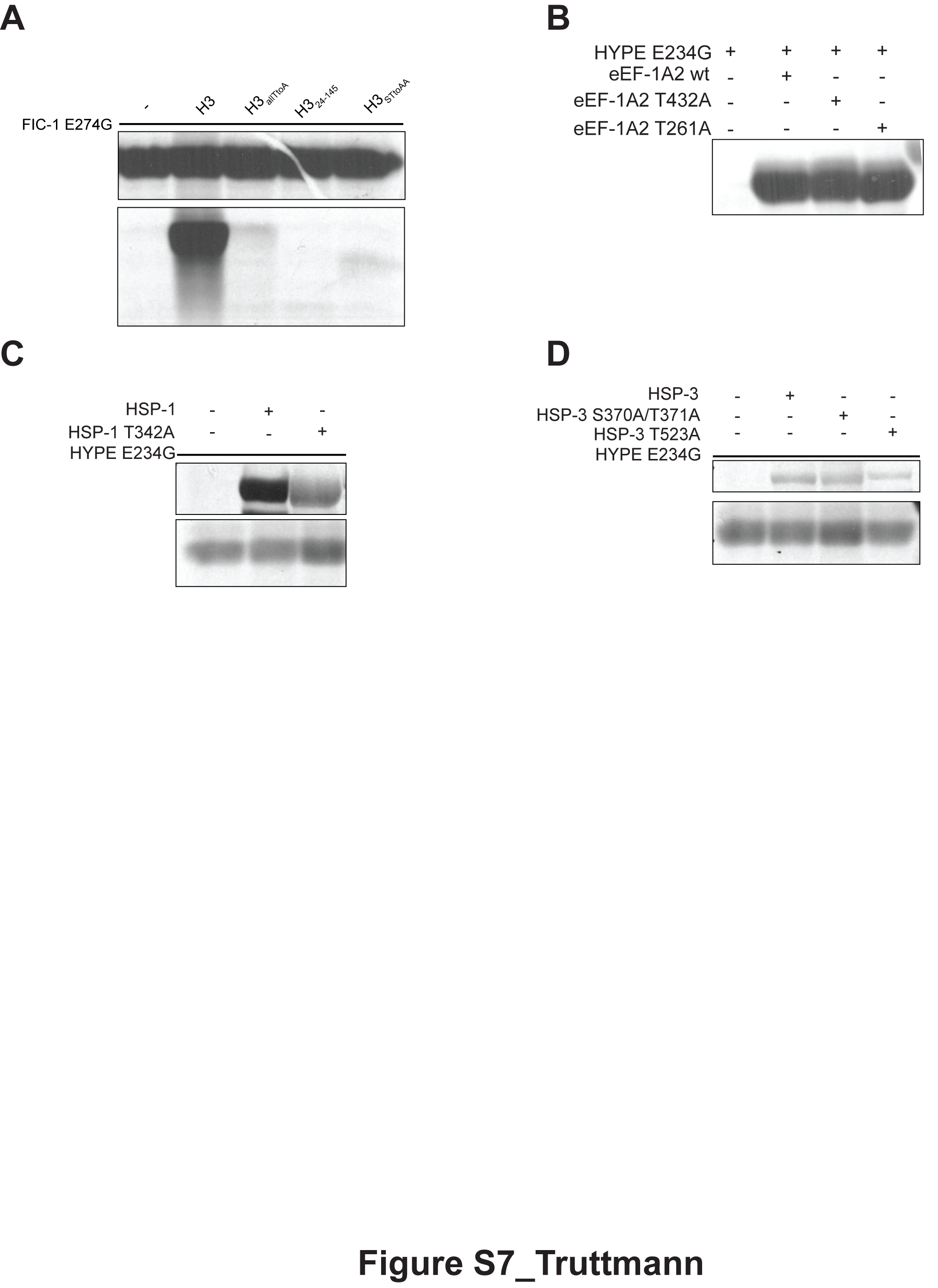

Supplement: S7 Fig — (A) FIC-1 AMPylates threonines on Histone H3: Recombinant FIC-1 E274G was incubated with α 33P-ATP for an hour at which point substrates (Histone H3 wild-type and mutants) were added and the mixture was incubated for an additional hour. Sample autoradiography was assessed. (B) HYPE modifies eEF-1A2: Recombinant HYPE E234G was incubated with α 33P-ATP for an hour at which point substrates (eEF-1A2244-463 wild type and mutants) were added and the mixture was incubated for an additional hour. Sample autoradiography was assessed qualitatively. (C-D) HYPE modifies HSP-1 and HSP-3 on distinct sites from human BiP: Recombinant HYPE E234G was incubated with α 33P-ATP for an hour at which point substrates (HSP-1, HSP-3 and respective mutants) were added and the mixture was incubated for an additional hour. Sample autoradiography was assessed. (TIF) [file pgen.1006023.s007.tif]

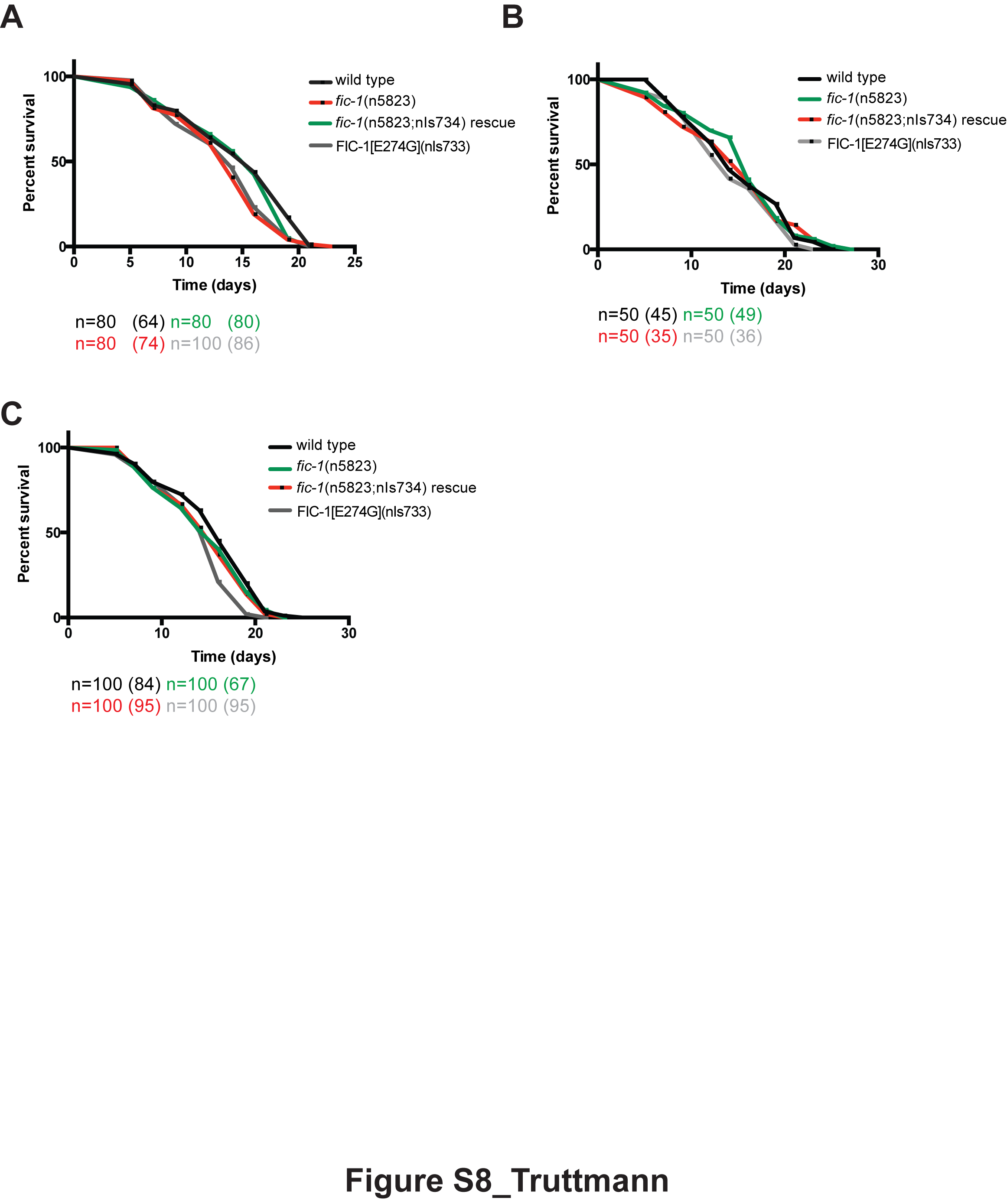

Supplement: S8 Fig — (A)—(C) wild type, fic-1(n5823), fic-1(n5823;nIs734) rescue and FIC-1[E274G](nIs733) animals were kept at either 20°C and survival was scored every other day. Depicted n refers to number of animals at experiment initiation; number in brackets represents total counted dead events. P-values (Gehan-Breslow-Wilcoxon test) as compared to N2 wild type control: (A) not significant; (B) not significant; (C) N2. vs fic-1(n5823, nIs734): 0.006; all others not significant. (TIF) [file pgen.1006023.s008.tif]

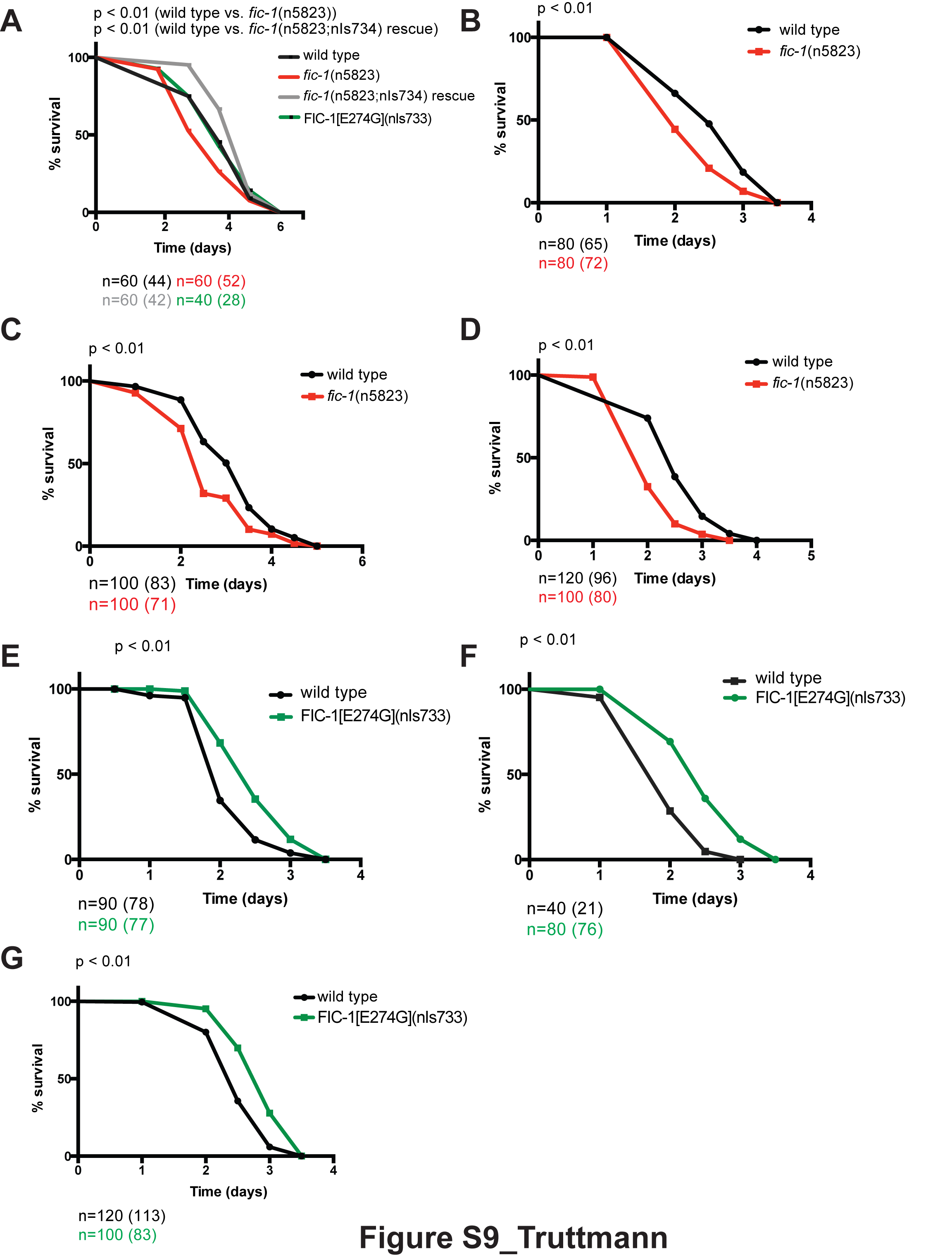

Supplement: S9 Fig — (A)—(G) wild type, fic-1(n5823), fic-1(n5823;nIs734) rescue and FIC-1[E274G](nIs733) L4 animals of indicted lines were place in the center of a P. aeruginosa loan and nematode survival was scored once per day until last animal vanished. Depicted n refers to number of animals at experiment initiation; number in brackets represents total counted dead events. P-values (Gehan-Breslow-Wilcoxon test) are as compared to N2 wild type control. (TIF) [file pgen.1006023.s009.tif]
