# Supplementary material for: The Caenorhabditis elegans Protein FIC-1 Is an AMPylase That Covalently Modifies Heat-Shock 70 Family Proteins, Translation Elongation Factors and Histones
Source: PLoS Genet. 2016 May 3;12(5):e1006023. doi: 10.1371/journal.pgen.1006023 (PMC4854385; doi:10.1371/journal.pgen.1006023)
Supplement: S1 Table — (PDF) [file pgen.1006023.s010.pdf]

Table S1: antibodies used in this study

**primary antibodies**			
provider	***cat.-No.***	***specificity***	***dilution used***
Santa Cruz Biotechnology	sc-805	HA (clone Y-11)	1:500 (IF); 1:5'000 (WB)
	-	Fic-1 serum	1:100 (IF); 1:1'000 (WB)
Cell Signaling	#3683	GAPDH (14C10)-HRP	1:1000 (WB)
GENETEX, INC	GTX113340	BiP/Grp78	1:3'000 (WB)
EMD Milipore	07-030	Histone H3 K4Met	1:3'000 (WB)
**secondary antibodies**			
provider	***cat.-No.***	***specificity***	***dilution used***
Qiagen	34460	penta-HIS	1:10'000
Fisher Scientific	OB4050-05	anti-rabbit IgG (H+L)	1:25'000
SouthernBiotech	1038-05	anti-mouse IgG (H+L)	1:25'000
