# Supplementary material for: The Caenorhabditis elegans Protein FIC-1 Is an AMPylase That Covalently Modifies Heat-Shock 70 Family Proteins, Translation Elongation Factors and Histones
Source: PLoS Genet. 2016 May 3;12(5):e1006023. doi: 10.1371/journal.pgen.1006023 (PMC4854385; doi:10.1371/journal.pgen.1006023)
Supplement: S2 Table — (PDF) [file pgen.1006023.s011.pdf]

Table S2: data collection and refinement statistics

Protein	Fic-1 130-508	Fic-1 (E274G) 130-508
**Organism**	*C. elegans*	*C. elegans*
**Data collection**		
Space group	R32	R32
a, b, c (Å)	192.2, 192.2, 182.5	193.0, 193.0, 182.7
α, β, γ (°)	90.0, 90.0, 120.0	90.0, 90.0, 120.0
Resolution range (Å)	123.0 – 2.9 (3.0 – 2.9)	96.5 – 3.7 (3.9 – 3.7)
Total reflections	176904	93499
Unique reflections	28518	13578
Completeness (%)	99.0 (97.6)[1]	100.0 (99.2)
Redundancy	6.3 (5.5)	6.9 (6.6)
R_{sym} (%)	17.1 (>100)	19.2 (91.5)
$R_{p.i.m.}$ (%)	8.8 (70.2)	7.7 (37.0)
I/σ	17.0 (1.3)	11.5 (2.0)
$CC_{1/2}$ (%)	99.6 (57.4)	99.7 (70.2)
**Refinement**		
Resolution range (Å)	123.0 – 2.9	96.5 – 3.7
R_{work} / R_{free} (%)	20.6 / 25.5	20.4 / 24.5
CC_{work} / CC_{free} (%)[2]	84.0 / 71.0	81.0 / 76.0
Coordinate error (Å)	0.39	0.46
Number of reflections		
Total	27933	13522
R_{free} reflections	1749	1348
Number of non-hydrogen atoms	5066	5066
Protein atoms	5056	5056
Water atoms	10	10
R.m.s. deviations		
Bond lengths (Å)	0.009	0.003
Bond angles (°)	1.462	0.674
Average B factors (Å^2)	68.2	109.4
Protein	67.0	109.4
Water	35.4	81.7
Ramachandran (%)		
Favored (%)	95.2	93.9
Allowed (%)	4.0	5.4
Outlier (%)	0.8	0.8

Clash score	9.6	9.0
MolProbity score	2.1	2.3
MolProbity percentile	98th	99th

[1] values in brackets are for the highest resolution shell comprising 10% of all data

[2] CC_{work}/CC_{free} are only reported for the highest resolution shell [1].

References

1. Diederichs K, Karplus PA. Better models by discarding data? Acta Crystallogr D Biol Crystallogr. 2013;69(Pt 7):1215-22. doi: 10.1107/S0907444913001121. PubMed PMID: 23793147; PubMed Central PMCID: PMCPMC3689524.
